# Metabolic Profiling for Detection of Staphylococcus aureus Infection and Antibiotic Resistance

**DOI:** 10.1371/journal.pone.0056971

**Published:** 2013-02-25

**Authors:** Henrik Antti, Anna Fahlgren, Elin Näsström, Konstantinos Kouremenos, Jonas Sundén-Cullberg, YongZhi Guo, Thomas Moritz, Hans Wolf-Watz, Anders Johansson, Maria Fallman

**Affiliations:** 1 Department of Chemistry, Computational Life Science Cluster, Umeå University, Umeå, Sweden; 2 Department of Molecular Biology, Umeå University, Umeå Centre for Microbial Research, Umeå, Sweden; 3 Department of Clinical Microbiology, Laboratory for Molecular Infection Medicine Sweden, Umeå Centre for Microbial Research, Umeå University, Umeå, Sweden; 4 Department of Medicine F59, Center for Infectious Medicine, Karolinska Institutet, Karolinska University Hospital, Stockholm, Sweden; 5 Division of Reproduction, Department of Clinical Sciences, Faculty of Veterinary Medicine and Agricultural of Sciences, SLU, Uppsala, Sweden; 6 Department of Forest Genetics and Plant Physiology, Swedish University of Agricultural Sciences, Umeå, Sweden; 7 Department of Molecular Biology, Laboratory for Molecular Infection Medicine Sweden, Umeå Centre for Microbial Research, Umeå University, Umeå, Sweden; Harvard Medical School, United States of America

## Abstract

Due to slow diagnostics, physicians must optimize antibiotic therapies based on clinical evaluation of patients without specific information on causative bacteria. We have investigated metabolomic analysis of blood for the detection of acute bacterial infection and early differentiation between ineffective and effective antibiotic treatment. A vital and timely therapeutic difficulty was thereby addressed: the ability to rapidly detect treatment failures because of antibiotic-resistant bacteria. Methicillin-resistant *Staphylococcus aureus* (MRSA) and methicillin-sensitive *S. aureus* (MSSA) were used *in vitro* and for infecting mice, while natural MSSA infection was studied in humans. Samples of bacterial growth media, the blood of infected mice and of humans were analyzed with combined Gas Chromatography/Mass Spectrometry. Multivariate data analysis was used to reveal the metabolic profiles of infection and the responses to different antibiotic treatments. *In vitro* experiments resulted in the detection of 256 putative metabolites and mice infection experiments resulted in the detection of 474 putative metabolites. Importantly, ineffective and effective antibiotic treatments were differentiated already two hours after treatment start in both experimental systems. That is, the ineffective treatment of MRSA using cloxacillin and untreated controls produced one metabolic profile while all effective treatment combinations using cloxacillin or vancomycin for MSSA or MRSA produced another profile. For further evaluation of the concept, blood samples of humans admitted to intensive care with severe sepsis were analyzed. One hundred thirty-three putative metabolites differentiated severe MSSA sepsis (n = 6) from severe *Escherichia coli* sepsis (n = 10) and identified treatment responses over time. Combined analysis of human, *in vitro*, and mice samples identified 25 metabolites indicative of effective treatment of *S. aureus* sepsis. Taken together, this study provides a proof of concept of the utility of analyzing metabolite patterns in blood for early differentiation between ineffective and effective antibiotic treatment in acute *S. aureus* infections.

## Introduction

The intensive use of antibiotics for 60 years has resulted in a challenge of global scale - the increasing rates of antibiotic resistance among bacteria. As a consequence, we are now running out of treatment options for many infections [1], [2], [3]. Methicillin resistant *Staphylococcus aureus* (MRSA) is a prime example of a common, global, and potentially dangerous pathogen that has acquired antibiotic resistance [2]. The current situation is a reminder of past times when little could be done to combat a *S. aureus* infection except surgical drainage of infected body sites. MRSA is resistant to all members of the β-lactam class of antibiotics including all penicillins, cephalosporins and carbapenems, thereby disarming all previous mainstay treatments against *S. aureus.* In addition, MRSA is frequently resistant to other common antimicrobial agents [4].

Worldwide, *S. aureus* is the most common cause of soft tissue, skin, and blood stream infection in humans both in society and hospital settings [4]. Localized disease may range in severity from harmless skin boils to severe pneumonia, endocarditis, surgical site infection, or osteomyelitis [5]. The bacterium may spread rapidly from these local sites into the blood stream causing severe sepsis. About 30% of patients diagnosed with *S. aureus* bacteremia die within 30 days [6], [7]. Due to increased frequency of MRSA, the first line treatment of suspected severe *S. aureus* infection has been shifted in many parts of the world from single drug treatment with penicillinase stabile penicillins to vancomycin, which specifically targets MRSA. Despite this, MRSA infections lead to a worse outcome with increased rates of fatality as compared to infections by methicillin susceptible *S. aureus* (MSSA) [1], [8].

Due to a lack of timely diagnostics, decisions by physicians regarding use of antibiotic therapy normally must be based on clinical symptoms without knowledge of the causative agent or its resistance status. Consequently, antibiotics with broad antimicrobial action are overused for maximizing treatment success. The evaluation of treatment response is also clinical and can at best be performed two days after treatment start. The over- and occasional misuse of antibiotics is continuously driving the resistance development among bacterial pathogens, and at times treatment failure remains undiscovered for several days due to an unexpected bacterial etiology or unforeseen resistance. For these reasons, new strategies to obtain rapid diagnostics as well as information on the response to treatment are needed.

In this study we have employed a metabolomics approach [9], [10] for studying *S. aureus* infection *in vitro,* in mice, and in humans. The objective was to develop a new concept for the early diagnosis of acute bacterial infection and antibiotic resistance. Samples from bacterial growth media, blood of infected mice and blood of humans with infection were analyzed with Gas Chromatography/Mass Spectrometry (GC/MS) followed by multivariate statistical analysis to reveal metabolic signatures of infection and the response to antibiotic treatment. Unique metabolic signatures were found for MSSA and MRSA infection during growth *in vitro*, as well as in the animal model. Moreover, effective antibiotic treatments could be distinguished from ineffective treatments at early time points. Finally, we assessed the metabolomic approach using samples from humans with severe MSSA sepsis and found that *S. aureus* sepsis was clearly distinguished from severe *E. coli* sepsis already at admission to an intensive care unit. This study provides a proof of concept for metabolomic methods using GC/MS for the early diagnosis of *S. aureus* infection, and determination of antibiotic resistance of the causative agent.

## Materials and Methods

### Ethics Statement

The animal experiments were approved by and performed according to the guidelines of the Swedish Ethical Committee on animal research in Umeå, Sweden (ethical permissions A81-08 and A90-11). The analysis of blood samples from humans were approved by the Regional Ethic Board at Karolinska University, Stockholm, Sweden and written informed consent was obtained from all participants in the study.

### Bacteria

The MRSA and MSSA strains used in this study were both from blood cultures processed in year 2009 with the instrumented BD Bactec Plus system at the Clinical Microbiology Laboratory at Umeå University Hospital, Umeå, Sweden. The MSSA strain was from a male with a surgical site infection in a shoulder and the MRSA strain from a male with a wound infection of a hand.

### Mice

Eight week old female BALB/c mice (Taconic, Denmark) were given normal mouse chow and water ad libitum, and were housed under standard conditions.

### Chemicals

All chemicals and compounds were of analytical grade unless stated otherwise. The 11 internal standards (IS) (isotope labelled) were purchased; [^2^H_7_]-cholesterol, [^13^C_4_]-disodium α-ketoglutarate, [^13^C_5_,^15^N]-glutamic acid, [1,2,3-^13^C_3_]-myristic acid, [^13^C_5_]-proline, and [^2^H_4_]-succinic acid from Cambridge Isotope Laboratories (Andover, MA, USA); [^13^C_6_]-glucose from Aldrich (Steinheim, Germany); [^13^C_4_]-palmitic acid (Hexadecanoic acid), [^2^H_4_]-butanediamine·2HCl (Putrescine), and [^13^C_12_]-sucrose from Campro (Veenendaal, The Netherlands); and [^2^H_6_]-salicylic acid from Icon (Summit, NJ, USA). Silylation grade pyridine and *N*-Methyl-*N*-trimethylsilyltrifluoroacetamide (MSTFA) with 1% trimethylchlorosilane (TMCS) were purchased from Pierce Chemical Co (Rockford, IL, USA). The stock solutions (reference compounds and IS) were all prepared in 0.5 µg/µL concentrations in either Milli-Q water or methanol.

### *In vitro* Growth

Three *in vitro* studies of bacterial growth were performed using complement inactivated human serum from three different donors. MRSA and MSSA bacteria were grown over night in LB (Lurea Broth) and grown in in 37°C with agitation, and the following day diluted to Optical Density (OD_600_) 0.02 in 80% human serum and 20% LB. At OD_600_ 0.2, 1 µg/ml Cloxacillin/Ekvacillin (Meda AB, Sweden) or 10µg/ml vancomycin (Axelia) were added to the cultures. Control samples were taken from cultures with no antibiotics added. Samples were taken 1, 2, and 4 h after the addition of antibiotics.

### Mouse Infections

Mice were infected intravenously (i.v.) with 1.2×10^6^ CFUs MRSA or 0.6×10^6^ or MSSA in 100 µl PBS. Control mice received an injection of 100 µl PBS. Both infected and control mice were given antibiotics i.v. 24 h post infection; 20 or 40 mg/kg Cloxacillin/Ekvacillin corresponding to 0.4 or 0.8 mg/mouse, or 110 mg/kg Vancomycin corresponding to 2.2 mg/mouse. Serum was sampled from the tail vein of all mice in the study prior to infection (which served as individual uninfected controls), 24 h post infection (p.i.), and at one, three, and six h after the addition of antibiotics. Serum was obtained by 30 min *clotting* at *room temperature, followed by centrifugation for* 10 min at 9400×*g*. Serum was immediately frozen and kept at −80°C.

### Patient Samples

The human serum samples used in this study were from two pooled prospective studies of severe sepsis and septic shock [11], [12]. The patients were enrolled at admission to the intensive care unit (ICU) of Karolinska University Hospital in Huddinge, a tertiary care facility in Sweden. The diagnoses of septic shock and/or severe sepsis were defined according to the criteria proposed by American College of Chest Physicians/Society of Critical Care Medicine [13]. For the current study, samples representing patients with blood culture confirmed *S. aureus* sepsis (N = 6; all males) or *Escherichia coli* sepsis (N = 10; 9 males and 1 female) were included. Venous blood was collected in vacutainer tubes (Becton Dickinson), was allowed 30 min clotting time after which it was centrifuged for 10 min at 1300×*g* in a swing bucket centrifuge; before serum separation and immediate freezing and storage at −80°C. Serum samples used in this study were obtained at admission, 24 h, 144 h, and after 2 weeks. Disease severity scores for the two groups of patients indicated severe disease at admission to the intensive care unit with median APACHE II score of 20.5 (mean 21; range 8–37) and 29.5 (mean 29.5; range 11–45) for the *S. aureus* and *E. coli* group respectively.

### Preparation of Samples for GC/MS Analysis

Samples were divided into batches for the analysis (both extraction/derivatization and GC/MS analysis). The batches were selected to include most of the between sample variation with regards to e.g. subject, time, infection and treatment. Extraction and derivatization of the samples were carried out according to the serum protocol for metabolomics available at Umeå Plant Science Centre (UPSC) [14] and was carried out in the same way for all included samples, although the volumes varied somewhat between the different studies. For the *in vitro* studies 100 µl samples were used, while in the mouse and human studies, 50 µl was used as start volumes for the extraction due to difficulties to obtain larger sample aliquots.

Volumes for the *in vitro* studies will be stated first followed by the mouse and human studies respectively in brackets; the same order is also used for the derivatization section below. For the extraction the frozen 100 µl (50 µl) aliquots of serum, in Eppendorf tubes (Sarstedt Ref: 72.690), were first thawed in room temperature and then put on ice. 900 µl (450 µl) methanol/water extraction mix (90∶10 v/v) including 11 IŚs (7 ng/µl), was added to all of the samples, still on ice, followed by extraction in a bead mill (MM 400, Retsch GmbH, Haan, Germany) for 2 min with a frequency of 30 Hz after which the samples were again put on ice (in a cold room 4–5°C) for 120 min. After the cooling the samples were centrifuged for 10 min at 14 000 rpm (Centrifuge 5417R, Eppendorf, Hamburg, Germany) at 4°C and then 200 µl (100 µl) of the supernatants were transferred to GC vials and evaporated to dryness using a speedvac (miVac, Quattro concentrator, Barnstead Genevac, Ipswitch, UK). After evaporation the samples were stored in −80°C until derivatization.

Prior to derivatization the extracted samples were run for 5–10 min in the speedvac to remove any possible condense. 30 µl (15 µl) methoxyamine in pyridine (15 µg/µl) was added followed by 10 min of vigorous shaking in a shaking machine and 60 min heating in an oven at 70°C. The reaction was then continued for 16 h in room temperature. 30 µl (15 µl) MSFTA (N-methyl-N-trimethylsilyl-trifluoroacetamide) +1% TMCS (Trimethylchlorosilane) was added to all samples for the trimethylsilylation step. The samples were then vortexed and the reaction time was set to 1 hour in room temperature. Finally, addition of 30 µl (15 µl) heptane including methyl stearate (15 ng/µl) was done followed by vortexing.

### GC/MS Analysis

GC/MS analysis was carried out based on the protocol presented by A et al [14]. One µl of each derivatized sample was injected splitless into an Agilent 6890 (Agilent, Atlanta, GA, USA) gas chromatograph using an Agilent 7683 auto sampler. The GC was equipped with a 10 m×0.18 mm×0.18 µm i.d. fused silica capillary column, chemically bounded to a 0,18 µm DB5-MS stationary phase column (J&W Scientific, Folsom, CA, USA). The injector temperature was 270°C and the purge flow was turned on after 60 sec with a rate of 20 ml/min. Helium was used as carrier gas with a flow rate through the column of 1 ml/min. Temperature programming was used with an initial column temperature of 70°C for 2 min, then the temperature increased with 40°C/min up to 320°C where it was held for 2 min. The effluent from the column was then led to the ion source of a Pegasus III-TOF-MS (Leco Corp., St Joseph, MI, USA) via a transfer line with a temperature of 250°C. The temperature of the ion source was 200°C. An electron beam of 70 eV was used to generate the electrons at an ionization current of 2.0 mA. 30 spectra/sec were recorded with masses in the range of 60 to 800 *m/z* until a solvent delay of 170 sec at which point the acceleration voltage was turned off. The detector voltage was 1500–1700 V. Apart from the study samples, several samples of methyl stearate in heptane (5 ng/µl) were run to check the sensitivity of the instrument and a few alkane series samples (containing C8–C40) were also run to allow calculation of retention indexes, RI. The analysis time for each sample was around 15 min.

### Data Processing

After GC/MS analysis the data was exported in two formats; SMP and NetCDF. MATLAB (7.3.0 R2006b and 7.11.0 R2010b, Mathworks, Natick, MA, USA) was used both for the pre-processing steps and for the curve resolution by means of hierarchical multivariate curve resolution (H-MCR) using an in-house developed script [15]. The pre-processing steps included the analysis of alkane series, background reduction, smoothing of data (filtering), alignment of chromatograms with a median sample as target, and division of chromatograms into time windows. The result of the curve resolution was a number of resolved chromatographic profiles (putative metabolites) together with corresponding mass spectra.

### Identification of Metabolites

NIST MS Search 2.0 was used to identify each resolved compound, by comparing the acquired spectra of these compounds with spectra from authentic standard compounds stored in mass spectral libraries. The libraries used for the identification were the in-house mass spectra library database established by UPSC or the mass spectra library maintained by the Max Planck Institute in Golm (http://csbdb.mpimp-golm.mpg.de/csbdb/gmd/gmd.html).

### Multivariate Data Analysis

Multivariate data analysis was performed using the computer software SIMCA (version SIMCA-P+12.0, Umetrics AB, Umeå, Sweden). Prior to multivariate analysis all data were mean centered (subtraction of the variable average for all individual variables) and scaled to unit variance (division of each variable with its standard deviation). Mean centering allows the multivariate models to merely focus on the inter-variable variation in the model interpretation. Scaling to unit variance implies that each variable is given the same chance to affect the model, which makes sense in a hypothesis generation screening approach as the one presented here. Principal component analysis (PCA) was used to get an overview of the data. Orthogonal partial least squares – discriminate analysis (OPLS-DA) was used to compare metabolic profiles of different sample classes. Depending on the outcome of the initial OPLS models additional models were calculated with altered sample class division. Since three different types of studies were included the strength of the models was investigated by trying to find common metabolites between the different studies and then build models based on only the common metabolites. This was initially performed by highlighting significant metabolites contributing to the separation of the classes from the OPLS-DA covariance loadings (w*) of similar models in each study (initial criteria for significance −0.04> w* >0.04). As a first filtering step only metabolites that were common in all three *in vitro* studies were used for comparison with the other studies. Metabolites showing different directions of change (increased or decreased levels) in the *in vitro* studies were excluded. The common *in vitro* metabolites were compared to the significant metabolites in the mouse and the human studies. Significant metabolites in the mouse study were also compared to the metabolites found in the human study. The final “common” metabolite pattern, being the combined metabolites common between *in-vitro* and human as well as between mouse and human was then evaluated in the human model. It should be noted that this “common pattern” could not be evaluated in the mouse and *in-vitro* model respectively since the individual contribution from those models not necessarily do overlap in terms of significant metabolites. The comparison of identified metabolites was performed mainly by comparing metabolite identities, but also similarities of mass spectra. Unidentified metabolites were compared by considering only mass spectra similarity. All multivariate models were visualized by score values from the extracted latent variables. For all OPLS-DA models, score values from the predictive components are shown, where t [1] p denote score values for the first component (model dimension) and t [2] p the second component. For the two-class models, cross-validated score values are shown (indicated as tcv [1] p). Models including predictions of independent samples are visualized using both cross-validated and predicted score values (indicated as tPS [1] cv [1] p). Cross-validated p-values, based on ANOVA of the cross-validated models, are given for all models to reveal the statistical significance of the class separation, where the significance limit was set to p<0.05.

### Quantification of Pattern Metabolites

Seven metabolite standards (serine, threonine, homoserine, ornithine, glutamine, myo-inositol and linoleic acid - all of which were of similar compound class to the possible biomarkers found) were used to generate calibration curves (at 6 different concentration levels) in order to quantify the possible biomarkers in the analyzed samples. Quantification was performed by calculating relative peak area response ratios (metabolite of interest peak area/internal standard peak area), after using two modes of metabolite confirmation: retention time similarity (mostly varied by a standard deviation less than 5%) and m/z spectra (similarity was usually greater than 700, where 1000 is a perfect match). Six internal standards were also present in every sample, namely: D4-succinic acid, 13C4-a-ketoglutarate, 13C5, 15N-glutamic acid, 13C4-hexadecanoic acid, 13C12-sucrose and D7-cholesterol. Quantification ions were used to perform the quantification for each metabolite of interest, as they provide more accurate peak area ratios when compared to TIC (total ion chromatogram) or *m/z* 73 (mass fragment produced from trimethylsilyl derivatization products) peak area ratios. Additionally, the ions monitored for quantification were most specific to the analyte of interest, provided a solid signal-to-noise (S/N) ratio and the least interference to other ions. RSDs and R^2^ values were all found to be within acceptable limits, with R^2^ values greater than 0.990 in almost all cases. The limit of detection (LOD) and limit of quantification (LOQ) were determined manually using the standards with the lowest concentration i.e. 0.01 mg/L and reconstructing the respective EIC for each of the seven metabolites of interest. Detection limits were calculated by determining the S/N ratio and extrapolating to the S/N = 3.3 level (for the LOD) and S/N = 10 level (for the LOQ). The LOD achieved using this method was 0.0005 mg/L and the LOQ attained was 0.001 mg/L for the seven standard metabolites. The LOQ was below the lowest standard, which is in agreement with the concentration range selected to construct the calibration curve. Table 1 titled ‘metabolic response to antibiotic treatment’ includes the concentration levels determined using the quantification method above in mg/L. The fold changes in concentration observed in the metabolites of interest ranged from two-fold to one-hundred fold, when comparing the two most obvious normal and infection samples (i.e. 0 h and 144 h).

**Table 1:** Metabolic response to antibiotic treatment.

Metabolite^a^	Change in concentrationwith effective treatment^b^	RI^c^	p-value	Concentration at0 h (mg/L)^d^	Concentration at144 h (mg/L)^d^
Glutamine	↑	1768	4.1×10^−2^	0.095	0.214
Homoserine	↑	1454	4.1×10^−2^	0.097	0.000
Inositol	↓	2080	5.8×10^−2^	0.314	0.099
Linoleic acid	↑	2207	5.5×10^−2^	0.182	0.003
Ornithine	↑	1610	2.2×10^−2^	0.047	0.062
Serine	↑	1363	7.7×10^−2^	0.045	0.008
Threonine	↓	1384	5.6×10^−2^	1.187	0.094

a Significant metabolites for *S. aureus* antibiotic treatment response common human *S. aureus* sepsis and mice infected with MRSA and MSSA, and human *S. aureus* sepsis and *in vitro* grown MRSA or MSSA.

b Refers to response to antibiotic treatment, where ↑/↓ indicates a higher/lower metabolite concentration in samples with effective treatment compared to samples with ineffective treatment (for *in vitro* experiments and mice infection) and in late time point, 144 h-2 weeks after admittance, compared to acute phase infection samples, 0–24 h after admittance (for human sepsis).

c Retention index for all metabolites.

d Concentration obtained from direct quantification of the 7 metabolites in a subset of the *S. aureus* samples shown both for samples in the acute phase (0 h) and in the late phase (144 h).

## Results

### Bacterial Growth *in vitro* and Response to Antibiotic Treatment Produces Unique Metabolomic Signatures

**Figure 1:**
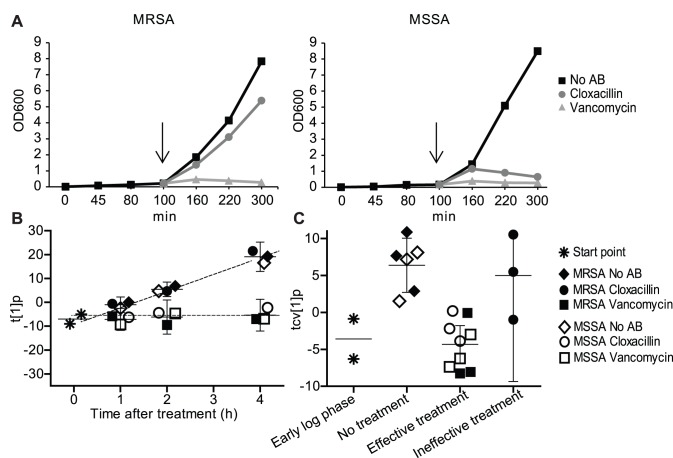
Metabolic profiles of samples from in vitro grown MRSA and MSSA in presence or absence of antibiotics. A) OD_600_ of MRSA and MSSA grown in absence of antibiotics or in presence of vancomycin, or cloxacillin. Arrows indicate time point for addition of antibiotics. B) OPLS-DA predictive score vector, t [1] p, for a seven class model based on 256 metabolites showing bacterial growth and response to antibiotic treatment over time. Mean score values with 95% CI are shown. The two regression lines represent the direction over time for the two metabolic responses. C) Cross-validated OPLS-DA predictive score vector, tcv [1] p, for a two class model based on 256 metabolites revealing a clear separation (p = 0.0013) between the two responses (effective and ineffective treatment). Mean score values with 95% CI are shown. The figure demonstrate the result from one out of three independent experiment (see Figure S1 and S2).

To evaluate if efficiency of antibiotic treatment can be measured by metabolic profiling of bacterial growth *in vitro*, we initially studied methicillin resistant and sensitive *S. aureus* (MRSA and MSSA, respectively) grown in presence or absence of cloxacillin or vancomycin. Overnight cultures of clinical isolates of MRSA and MSSA were diluted to OD_600_∼0.02 in 80% human heat inactivated serum with 20% LB and grown to OD_600_∼0.2 (indicated by an arrow; Figure 1A) when each strain was subdivided into three separate cultures; non-treated bacteria for continued growth, vancomycin treated bacteria, and cloxacillin treated bacteria. Both strains were sensitive to vancomycin, which served as a positive control for inhibition of growth, and translates to effective treatment of infection in a clinical setting. MSSA growth was successfully inhibited by cloxacillin (effective treatment), while the growth of methicillin resistant MRSA in cloxacillin was not inhibited (ineffective treatment) (Figure 1A). The metabolic profiles were determined for samples taken at different time points during the culturing (Figure 1B). Samples were taken during growth with or without antibiotics. Early log phase samples (OD_600_ 0.2) were used as a reference for untreated bacteria (Figure 1B). There were 256 putative metabolites detected by GC/MS analysis. Using multivariate analysis, we observed differentiating metabolite patterns for effective and ineffective treatment (Figure 1B). MRSA treated with cloxacillin (ineffective treatment) displayed the same patterns as untreated MRSA or MSSA at each time point, indicating metabolites associated with growing *Staphylococci*. In contrast, MRSA treated with vancomycin and MSSA treated with cloxacillin or vancomycin (effective treatment) displayed patterns consistent with inhibited growth (effective treatment). Furthermore, to explore the significance of the difference between the two responses, and to identify significantly altering metabolites, a multivariate model was calculated comparing samples belonging to either of the two responses (Figure 1C). The comparison revealed a significant separation between the two classes/responses (p = 0.0013). A separation could be seen already from 1 h after onset of treatment. We performed three independent experiments (using serum from three different blood donors) that were analyzed separately. These separate analyses showed consistent response profiles, thus validating the metabolic profile (Figure 1B, 1C; and Figure S1 and S2). In total, 32 significantly discriminating metabolites (26 identified and 6 unidentified) were altered between effective and ineffective treatment according to the model in all three experiments (Table S1). Thus, we could discriminate between growing and non-growing bacteria by using metabolic profiling. Since bacteria sensitive to antibiotics stop proliferation while resistant strains continue to grow, treatment efficiency by different antibiotics could be monitored.

### Unique Metabolic Profiles of Effective and Ineffective Antibiotic Treatment of *S. aureus* in Mice

**Figure 2:**
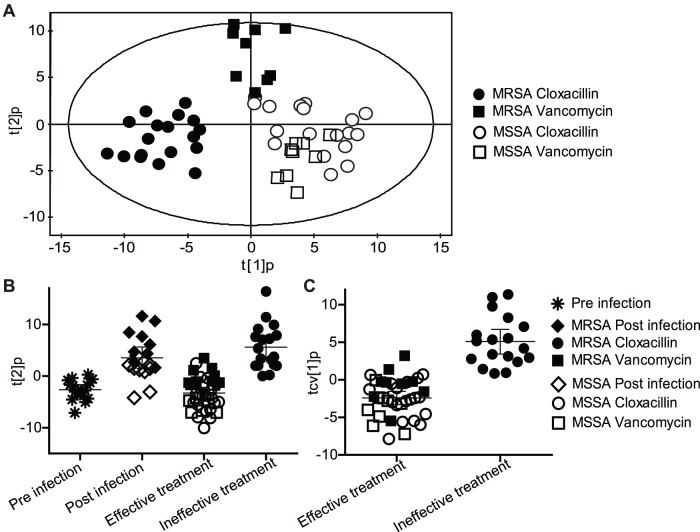
Metabolic profiles of serum from mice infected with *S. aureus*. A) OPLS-DA score plot (predictive score vectors t [1] p vs. t [2] p) for a four class model based on 474 metabolites showing metabolic profiles of MRSA or MSSA infected mice treated with cloxacillin or vancomycin. The first and second model components reveal discrimination between the different treatment groups. B) OPLS-DA predictive score vector, t [2] p, for a seven class model based on 474 metabolites separating the metabolic responses in mice in response to effective or ineffective antibiotic treatment. Mean score values with 95% CI are shown. C) Cross-validated OPLS-DA predictive score vector, tcv [1] p, for a two class model based on 474 metabolites for biomarker detection (p = 4.2 * 10^−7^) between ineffective versus effective treatment, i.e. MRSA treated with cloxacillin (ineffective treatment) versus MSSA and cloxacillin and MRSA/MSSA and vancomycin (effective treatment). Mean score values with 95% CI are shown.

To evaluate infection associated metabolic profiles *in vivo*, BALB/c mice were infected with MRSA or MSSA. Serum was sampled from all mice before infection, at 24 h p.i. (but preceding treatment with cloxacillin or vancomycin), and at 1, 3, and 6 h after antibiotic treatment. Using GC/MS, we were able to detect 474 putative metabolites. Multivariate analysis revealed a distinct metabolic pattern associated with ineffective antibiotic treatment (p = 0.00097), i.e. the metabolic profile of samples from MRSA-infected mice treated with cloxacillin was clearly different compared to the others (Figure 2A). Analysis of effective versus ineffective (MRSA and cloxacillin) antibiotic treatment revealed distinct metabolite profiles (Figure 2B). In accordance with the data from the in vitro analyses, the samples from untreated mice (24 h p.i.) co-varied with the samples of ineffectively treated mice (MRSA and cloxacillin), i.e. they expressed similar metabolic profiles, indicating bacterial growth. In contrast, samples from uninfected mice (pre-infection in Figure 2B) co-varied with samples of effectively treated mice (MSSA and cloxacillin, and MRSA/MSSA and vancomycin). This indicates that metabolic profiling is able to measure success or failure of treatment *in vivo*. To explore the statistical significance of the difference between the two responses, and also to identify potential diagnostic biomarkers, a model was calculated comparing samples from effectively and ineffectively treated mice (Figure 2C). The comparison revealed a separation between the two classes/responses (p = 4.2×10^−7^) (Figure 2C). In total 167 significantly discriminating metabolites (58 identified and 109 unidentified), either significantly increased or decreased, were detected in the two class model (Table S2). A comparison of these with the results of the previous *in vitro* experiments revealed 9 metabolites (7 identified, 2 unidentified) as common markers for effective treatment (Table S3). Thus, it seems feasible to identify common metabolites that indicate treatment efficiency in both *in vitro* and *in vivo* experimental infection systems.

### Metabolic Profiling of Human Severe Sepsis Caused by *S. aureus* or *E.coli*

**Figure 3:**
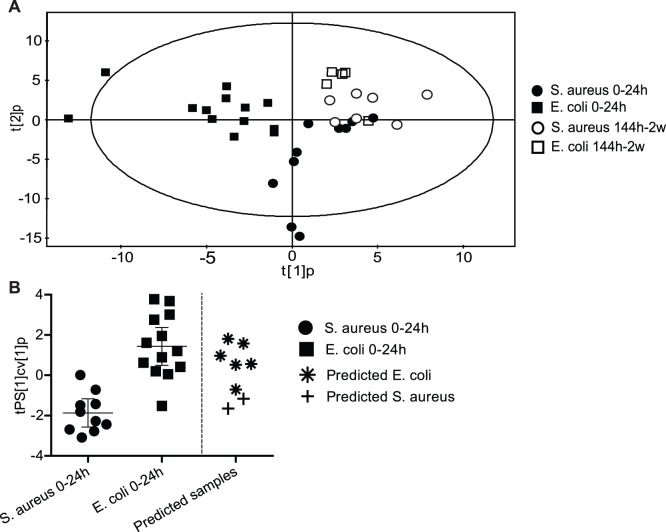
Discrimination between the metabolic profiles of *S. aureus* and *E. coli* sepsis in humans and effect of antibiotic treatment. A) OPLS-DA predictive score plot (score vectors t [1] p vs. t [2] p) for a four class model based on 228 metabolites revealing discrimination of severe sepsis caused by *S. aureus* or *E. coli* in the acute phase. B) Cross-validated and predicted OPLS-DA predictive score values, tPS [1] cv [1] p, for a two class model, based on 37 metabolites for discrimination of *S. aureus* and *E. coli* infection (p = 0.052). Out-of-sample prediction of sera of four patients at two time points. Mean score values with 95% CI are shown.

In order to establish if metabolic profiling of blood can be used to distinguish severe *S. aureus* sepsis from severe *E. coli* sepsis in humans, and to evaluate the effect of antibiotic treatment, we analyzed clinical samples obtained at an intensive care unit (ICU). All patients received effective antibiotic treatment at admission (i.e. β-lactams like cephalosporin and carbapenems). The causative *S. aureus* or *E. coli* was subsequently tested susceptible to the antimicrobial agents used for treatment. For metabolomic analyses, samples taken at the arrival to the intensive care unit (0–24 h) and samples taken after treatment (144 h –2 weeks) were employed. Metabolic profiling revealed 228 putative metabolites detected by GC/MS analysis. Using multivariate analysis we observed differentiating metabolite patterns between *S. aureus* and *E. coli* samples in the acute phase of disease (0–24 h; Figure 3A). The treatment responses were indeed reflected in the metabolic profiles with convergence over time (Figure 3A). At the late time points 144 h –2 weeks after admission, the profiles of *S. aureus* and *E. coli* clustered consistently with successful treatment of both infections. To verify that metabolomics can be used to distinguish *S. aureus* and *E. coli* sepsis, a multivariate model for the difference between *S. aureus* and *E. coli* samples in the acute phase of disease (0–24 h after admittance) was calculated based on the 228 metabolites. The model detected and revealed a clear separation based on a pattern of 133 significantly discriminating metabolites (52 identified and 81 unidentified) (model not shown).

### A Diagnostic Pattern for Severe Sepsis Caused by *S. aureus*

The availability of human samples of severe sepsis allowed us to use *E. coli* samples as controls for establishing a *S. aureus* specific diagnostic pattern. Out of the 228 metabolites used in the two-class model for separating early *E. coli* and *S. aureus* sepsis in humans, 133 were significant for the separation. Comparison of the 133 metabolites with the significant metabolites seen in ineffectively or untreated *S. aureus* infections *in vitro* and in mice identified 33 metabolites as characteristic for *S. aureus* infection. When a model was made on the 33 metabolites, this *S. aureus*-associated metabolic profile significantly discriminated acute *S. aureus* from *E. coli* infection in the human samples (p = 0.052) (Figure 3B, Table S4). Furthermore, this model could also be used for out-of-sample prediction of sepsis caused by *S. aureus* in comparison to *E. coli*. Importantly, eight samples of human sepsis were correctly classified to originate from *S. aureus* or *E. coli* sepsis (Figure 3B).

**Figure 4:**
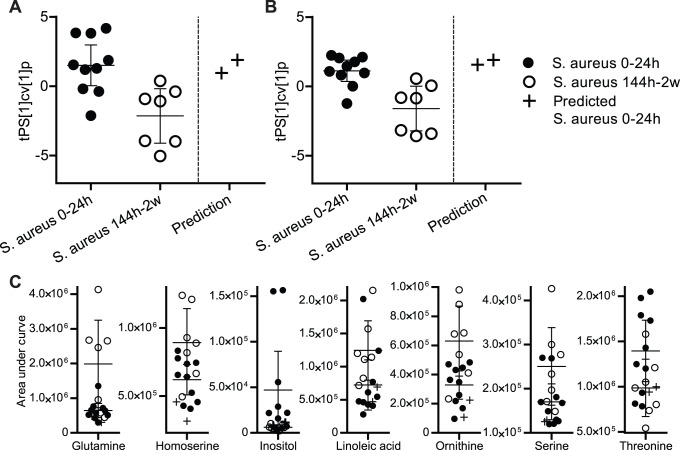
Monitoring the effect of antibiotic treatment response in *S. aureus* infection. A) Cross-validated and predicted OPLS-DA predictive score values, tPS [1] cv [1] p, for a two class model, based on 25 metabolites for discrimination of early and late *S. aureus* infection (p = 0.069). Out-of-sample prediction of sera of one patient from two time points. Mean score values with 95% CI are shown. B) Cross-validated and predicted OPLS-DA predictive score values, tPS [1]c v [1] p, for a two class model based on 7 metabolites for discrimination of early acute and late *S. aureus* infection (p = 0.0022). Out-of-sample predictions of sera of one patient at two time points. C) Relative concentration, determined as area under curve, for seven metabolites constituting the predictive pattern for treatment response in *S. aureus* sepsis samples. Errors bars represent 95% confidence intervals around the class average.

To further explore if metabolomics can detect antibiotic treatment effect in human samples, a two class model for the difference between acute and antibiotic treated *S. aureus* infection (0–24 h vs 144 h - 2 weeks after admittance) was calculated based on the 228 metabolites. The separation was based on 126 metabolites that were significantly altered, of which 52 could be assigned with a molecular identity. By comparing significantly altered metabolites from human and *in vitro* samples, and from human and mice samples, 25 common metabolites indicating effective antibiotic treatment for *S. aureus* sepsis were found (p = 0.069) (Figure 4A, Table S5, S6, and S7). Moreover, this profile could also be used to predict (diagnose) unknown samples from one patient with *S. aureus* sepsis, i.e. this patient was correctly classified as having acute infection, whereas samples from *E. coli* sepsis did not fit into the *S. aureus* model (p (t-test) = 0.004 and p (F-test) = 0.0013). Finally the strongest metabolic pattern among the 25 metabolites was determined by finding the combination of metabolites yielding the best classification model. This resulted in a pattern of 7 metabolites (Table 1). This pattern was used to explore the possibility to monitor effective treatment of *S. aureus* (p = 0.0022) (Figure 4B). Using this pattern, out-of-sample prediction of acute *S. aureus* infection was again correct (Figure 4B, compare with Figure 4A). The relative concentrations of each of the seven included metabolites (Figure 4C) clearly demonstrate that it is the combination of the metabolites in a metabolic pattern that is important to correctly monitor the treatment responses caused by the infection, not single metabolite concentrations. Targeted quantification of the seven metabolites using GC/MS was carried out in a subset of the *S. aureus* samples (N = 4; two acute and two late *S. aureus* samples) and verified the expected differences in concentration for the metabolites: glutamine, inositol, ornithine, and threonine, while the concentration changes of homoserine, linoleic acid, and serine were not verified (Table 1). However, use of the seven targeted concentration levels as a pattern resulted in significant separation between acute and antibiotic treated *S. aureus* infection in a two class model (p = 0.00048, Student’s t-test of the cross validated model scores).

## Discussion

In an era that was recently denoted “post-antibiotic” by the Director-General of the World Health Organization, new concepts for diagnostics of infectious disease and antibiotic resistance are needed [16]. New approaches that allow for more precise selection and adjustments of antibiotic treatment regimens could improve the outcome of severe infections caused by antibiotic resistant bacteria. First, a diagnostic test that can determine if the administrated antibiotic treatment of *S. aureus* is effective within a few h of initiation could help in optimize the antimicrobial therapy. Second, early etiologic diagnosis could help to reduce use of unnecessary antibiotic treatments that further exaggerates the resistance development and spread among pathogens. With current diagnostic procedures, it remains unknown if an infection is caused by MSSA or MRSA during at least two days until a culture diagnosis including analysis of antibiotic resistance is completed. Taken together there is a pressing need for improved diagnostic tests that are faster and can rapidly measure the success or failure of antibiotic treatment.

In the current study we have explored the potential of using metabolomics in the diagnosis of acute *S. aureus* infection and as a tool to measure success or failure of antibiotic treatment *in vitro* and *in vivo*. Our results are consistent with results from other disciplines in medical science which have found that metabolomic profiling of small biomolecules in body fluids can be used to understand metabolic events resulting from different disease and physiological conditions [17], [18], [19], [20]. We believe that metabolomics of infections is a promising and emerging approach for diagnostic purposes, in the early detection of treatment success or failure, and for obtaining an increased understanding of bacterial pathogenesis [21], [22]. Metabolomic analyses of culture supernatants from *S. aureus* grown *in vitro* could discriminate MRSA from MSSA, which is likely due to clonal variation. However, the important finding was that we could monitor efficiency of the cloxacillin treatment with this method. Similarly, analysis of blood samples from an experimental infection model in mice using the same bacterial strains showed that this discrimination was possible also from samples of an *in vivo* infection model. The results showed that it was possible to determine if the bacteria continued to replicate or if their replication was halted by effective antibiotic treatment *in vitro* and *in vivo*. Moreover, by comparing the metabolic profile indicating effective treatment for the *in vitro* MRSA/MSSA experiments and the animal experiments, metabolites that were shared between these infection model systems could be identified. A metabolic profile of nine metabolites useful for predicting successful antibiotic treatment was common between the *in vitro* and *in vivo* experiments. We believe that the existence of shared metabolites indicates a bacterial role for their alteration, either that the metabolites originate from the bacteria itself or from bacteria-induced host processes.

Analysis of severe human sepsis assessed only MSSA infections and not MRSA infection since MRSA is exceedingly rare in Sweden - the location of this study (less than one percent of positive *S. aureus* blood cultures). Nevertheless, the analyses showed that severe *S. aureus* sepsis was readily distinguished from severe *E. coli* sepsis. The difference in metabolomic profiles was largest at admission to the ICU, after which profiles in those who survived the sampled period (14/16) converged over time. The most obvious interpretation of this finding is that we have been able to measure the effect of effective antibiotic treatment of both disease conditions. The distinct metabolic profiles observed at admission to the ICU were specific for *S. aureus* and *E. coli*, respectively. As the result of effective antibiotic treatment the metabolite profiles then converged to reflect a recovery from infection shared between patients admitted with either *S. aureus* or *E. coli*. Taken together the results indicate that metabolic responses are specific to *S. aureus* or *E. coli* infections and that effective antibiotic treatment can be measured in specimens from patients.

Based on these results we strongly believe that single metabolites will not be sufficient as biomarkers for infection and effective antibiotic treatment. Instead, we suggest that a unique metabolic profile or “pattern” of a number of indicative metabolites is the important tool for diagnosis. We found that by using a pattern of seven serum metabolites it was possible to obtain a significant model, i.e. a latent variable, useful for predicting effective treatment in humans with severe *S. aureus* sepsis. By evaluating the seven metabolites individually it was evident that none of them alone provided the same level of significance as the combined pattern. This highlights the value of taking into account the correlation structure between variables to obtain a stronger and more robust discriminating pattern, i.e. a latent variable comprising of multiple inter-correlated and co-varying entities. We validated the robustness and the significance of the latent variable both by using a cross-validation procedure and by blind predictions of samples not included in the model calculations. By using the cross-validated model as a means to decide the significance we aimed at avoiding the risk of generating an over-fitted (false positive) model, which is a common criticism of multivariate approaches used in biological and medical sciences [9], [10]. In addition, this approach made it possible to obtain a fair estimation of the significance of the latent variable for discriminating the sample classes. Finally, by making blind predictions based on the extracted metabolite pattern we could test its diagnostic potential.

This study is descriptive in nature and we acknowledge that interpretation of the biological meaning of metabolite changes will be speculative and can mainly serve for generation of hypotheses. We discovered a general pattern of increased amino acid concentrations with effective treatment. Four out of five amino acids in the seven metabolite pattern (glutamine, homoserine, ornithine, and serine) increased in approximate concentration levels in response to effective treatment of *S. aureus* infection. Catabolism and muscle wasting are well known to occur during severe sepsis and it is generally believed that sepsis creates an energy deficit and that amino acids are consumed during sepsis for energy production by increased gluconeogenesis in the liver. It is possible that we have detected a switch away from such a catabolic state resulting from effective treatment. However, since the metabolic response was also detected under *in vitro* conditions, and the pattern of amino acid consumption differed between *S. aureus* and *E. coli* sepsis in humans, we hypothesize that the consumption is not only a host related response to sepsis, but is also mediated by the bacteria. Exoproteases are well-known virulence factors of *S. aureus* with a main function of converting local host tissue into nutrients required for bacterial growth, making it plausible that we are measuring an effect of *S. aureus* proteases degrading human tissue for bacterial nutrition. We posit that the increased levels of four amino acids in response to treatment may be a reflection of inhibited *S. aureus* growth and amino acid consumption and that there was a time lag before the activity of bacterial proteases ceased. We acknowledge that this does not explain why threonine levels decreased rather than increased. However, the overall pattern found was increased levels of amino acids.

We also found linoleic acid to be increased in response to effective treatment of *S. aureus* infection. This makes biological sense since linoleic acid is a free fatty acid with an anti *S. aureus* effect used by the host for infection defense [23], [24]. Linoleic acid is believed to be an important part of the local host defense of the skin towards gram positive pathogens such as *S. aureus,* and our finding of elevated levels in *S. aureus* infection therefore would fit with a host defense role of this metabolite in *S. aureus,* but not *E. coli* infection.

Finally, the decrease in inositol levels upon effective treatment in mouse and human samples but not in *in vitro* samples suggests that the observation is dependent on the interaction between the bacterium and the host. Inositol is a sugar alcohol and an important building stone of several secondary messengers in eukaryotic cells, mediating intracellular signal transduction [25]. Whether the high inositol values in the acute phase (or during ineffective treatment of mice) is related to the growth of *S. aureus* in the host or is a response to heavy tissue destruction causing release of inositol from damaged host cells is unclear. The *S. aureus* genome contains a putative gene encoding inositol monophosphatase that should be capable of dephosphorylating inositol phosphate to inositol. The gene may have a role in biofilm formation but the knowledge of inositol in bacteria is generally scarce 26,27.

The current study was designed to evaluate metabolic techniques for diagnosing *S. aureus* infection in three different settings, each with its distinct advantages and disadvantages. The target was the identification of metabolites corresponding to MSSA and MRSA metabolism during antibiotic treatment. The first setting included *in vitro* experiments. These avoided the complication of simultaneously measuring both host and bacterial metabolism. Although they said little or nothing about host response, they offered controlled experimental conditions, which allowed exploration of metabolomic changes during treatment of *S. aureus*. In the second setting, the complexity of host response was added to the mix, in the form of a mice model. Although mouse-specific metabolic host responses were not our primary interest, murine experiments provided opportunities to again perform controlled trials with genetically identical mice, defined doses of the same bacterial strains and consistent use of the same antibiotic treatments. Such an approach is not possible in a clinical patient trial. The third setting was human infection. The main disadvantage in our human model was a lack of MRSA infections (due to low incidence in Sweden, where this study was performed), which precluded a direct comparison with MSSA infections. Despite this, we consider the analysis of human samples a distinct strength of this study because; a) they are typical of real clinical situations and; b) we have included control samples from patients with *E. coli* sepsis. The controls help in identifying/discriminating not only metabolites of severe sepsis but more specifically those of severe sepsis caused by *S. aureus* infection. Future studies should include samples of humans infected with MRSA.

Although we could detect a relatively high number of putative metabolites in the different models using GC/MS for metabolic characterization, we noted some limitations with regards to identification of these compounds as well as in the sensitivity of the method. GC/MS is considered to be fairly straightforward in the comprehensive identification of metabolites, by using both available mass spectral libraries (e.g. NIST08 library) and libraries built by using authentic standard compounds (in-house libraries). However, although these libraries are continuously being updated, they are still far from being complete, resulting in a majority of detected putative metabolites remaining unidentified (in this study ∼60% of the detected peaks remained unidentified). It was also evident from our analysis that the GC/MS method has limitations due to the need of derivatization, and thereby the inborn discrimination of metabolites detected. For future studies we believe that the use of complementary, and for many types of metabolites, a more sensitive method such as liquid chromatography – mass spectrometry (LC-MS) will be of great value. An alternative is to use a more targeted approach, where a sub-set of pre-defined metabolites or compound classes are quantified with more accurate and precise mass spectrometric methods as well as increased sensitivity.

### Conclusion

In conclusion our results point toward a new way of thinking regarding identification of biomarkers for point of care diagnostics in the future. The idea of using metabolite patterns for diagnostics makes sense since the use of single molecular markers rarely results in the demanded specificity. By using mathematical modeling metabolic patterns can be described and explain how large numbers of metabolites are correlated in a complex biological system. Here, a direct analogy can be made with making a clinical diagnosis; something that usually requires a combination of many different signs, symptoms, facts in a clinical history, and laboratory tests before a synthesis (a model of disease) points to the correct diagnosis. The rise in antibiotic resistance levels among bacteria is increasing the need for rapid recognition of effective or ineffective antibiotic treatment at early stages of infection, something that underscores the value of the metabolomics approach. Future plans include speeding up analysis to enable early monitoring of treatment. This would constitute a significant improvement with the potential to reduce mortality from severe infections. Importantly, a rapid and specific diagnostic method for infection would also reduce use of unnecessary broad spectrum antibiotics. Although much work remains before this approach can be put into clinical practice, the results of this study provide a proof of concept for metabolomics as a tool for detecting putative biomarkers for antibiotic resistance.

## Supporting Information

Figure S1 **Metabolic profiles during in vitro growth of MRSA and MSSA.** A) OD_600_ of MRSA and MSSA grown in absence of antibiotics, with vancomycin, or cloxacillin. Arrows indicate time point for addition of antibiotics. B) OPLS-DA predictive score vector, t [1] p, for a seven class model based on 237 metabolites showing bacterial growth and response to antibiotic treatment over time. Mean score values with 95% CI are shown. The two regression lines represent the direction over time for the two metabolic responses. C) Cross-validated OPLS-DA predictive score vector, tcv [1] p, for a two class model based on 237 metabolites (p = 0.027) between the two responses. Mean score values with 95% CI are shown.
(TIF)

Figure S2 **Metabolic profiles during in vitro growth of MRSA and MSSA.** A) OD600 of MRSA and MSSA grown in absence of antibiotics, with vancomycin, or cloxacillin. Arrows indicate time point for addition of antibiotics. B) OPLS-DA predictive score vector, t [1] p, for a seven class model based on 367 metabolites showing bacterial growth and response to antibiotic treatment over time. Mean score values with 95% CI are shown. The two regression lines represent the direction over time for the two metabolic responses. C) Cross-validated OPLS-DA predictive score vector, tcv [1] p, for a two class model based on 367 metabolites revealing discrimination (p = 0.035) between the two responses. Mean score values with 95% CI are shown.
(TIF)

Table S1 **Individual metabolite response to antibiotic treatment** ***in vitro*** **.**
(DOCX)

Table S2 **Individual metabolite response to antibiotic treatment** ***in vivo*** **.**
(DOCX)

Table S3 **Individual metabolite response to antibiotic treatment common between** ***in vitro*** **grown MRSA and MSSA and mice infected with MRSA and MSSA.**
(DOCX)

Table S4 **Metabolite changes of** ***S. aureus*** **infection that is common between samples from humans, mice, and** ***in vitro*** **.**
(DOCX)

Table S5 **Individual metabolite response common between** ***in vitro*** **grown MRSA and MSSA and human** ***S. aureus*** **sepsis.**
(DOCX)

Table S6 **Individual metabolite response common between mice infected with MRSA and MSSA and human** ***S. aureus*** **sepsis.**
(DOCX)

Table S7 **Individual metabolite response common between human** ***S. aureus*** **sepsis and mice infected with MRSA and MSSA, and human** ***S. aureus*** **sepsis and in vitro grown MRSA and MSSA.**
(DOCX)

Table S8 **Summary of characteristic and high intensity m/z values for the different sets of common metabolites.**
(DOCX)
