# Supplementary figures and images for: Metabolic Profiling for Detection of Staphylococcus aureus Infection and Antibiotic Resistance
Source: PLoS One. 2013 Feb 25;8(2):e56971. doi: 10.1371/journal.pone.0056971 (PMC3581498; doi:10.1371/journal.pone.0056971)

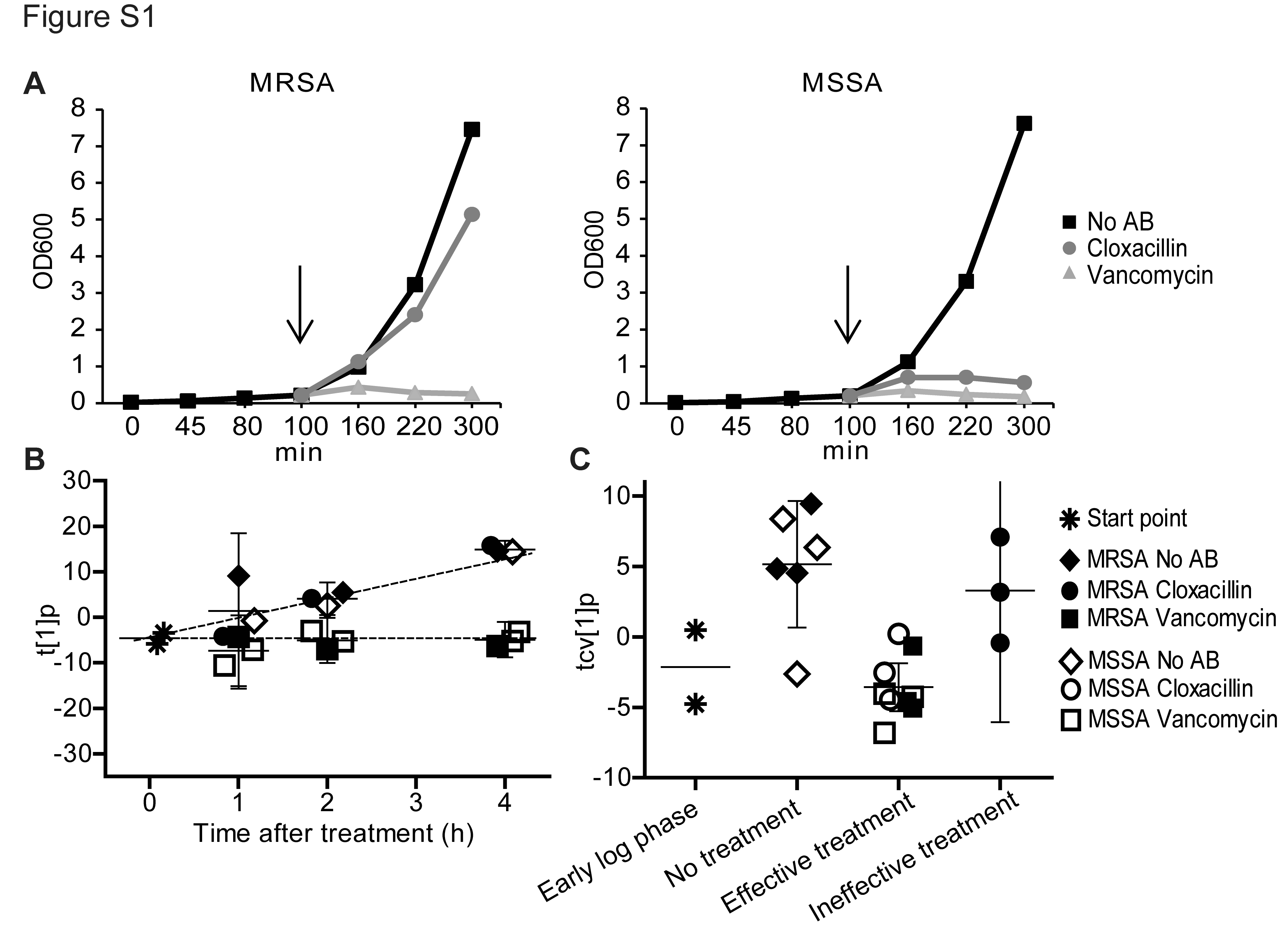

Supplement: Figure S1 — Metabolic profiles during in vitro growth of MRSA and MSSA. A) OD600 of MRSA and MSSA grown in absence of antibiotics, with vancomycin, or cloxacillin. Arrows indicate time point for addition of antibiotics. B) OPLS-DA predictive score vector, t [1] p, for a seven class model based on 237 metabolites showing bacterial growth and response to antibiotic treatment over time. Mean score values with 95% CI are shown. The two regression lines represent the direction over time for the two metabolic responses. C) Cross-validated OPLS-DA predictive score vector, tcv [1] p, for a two class model based on 237 metabolites (p = 0.027) between the two responses. Mean score values with 95% CI are shown. (TIF) [file pone.0056971.s001.tif]

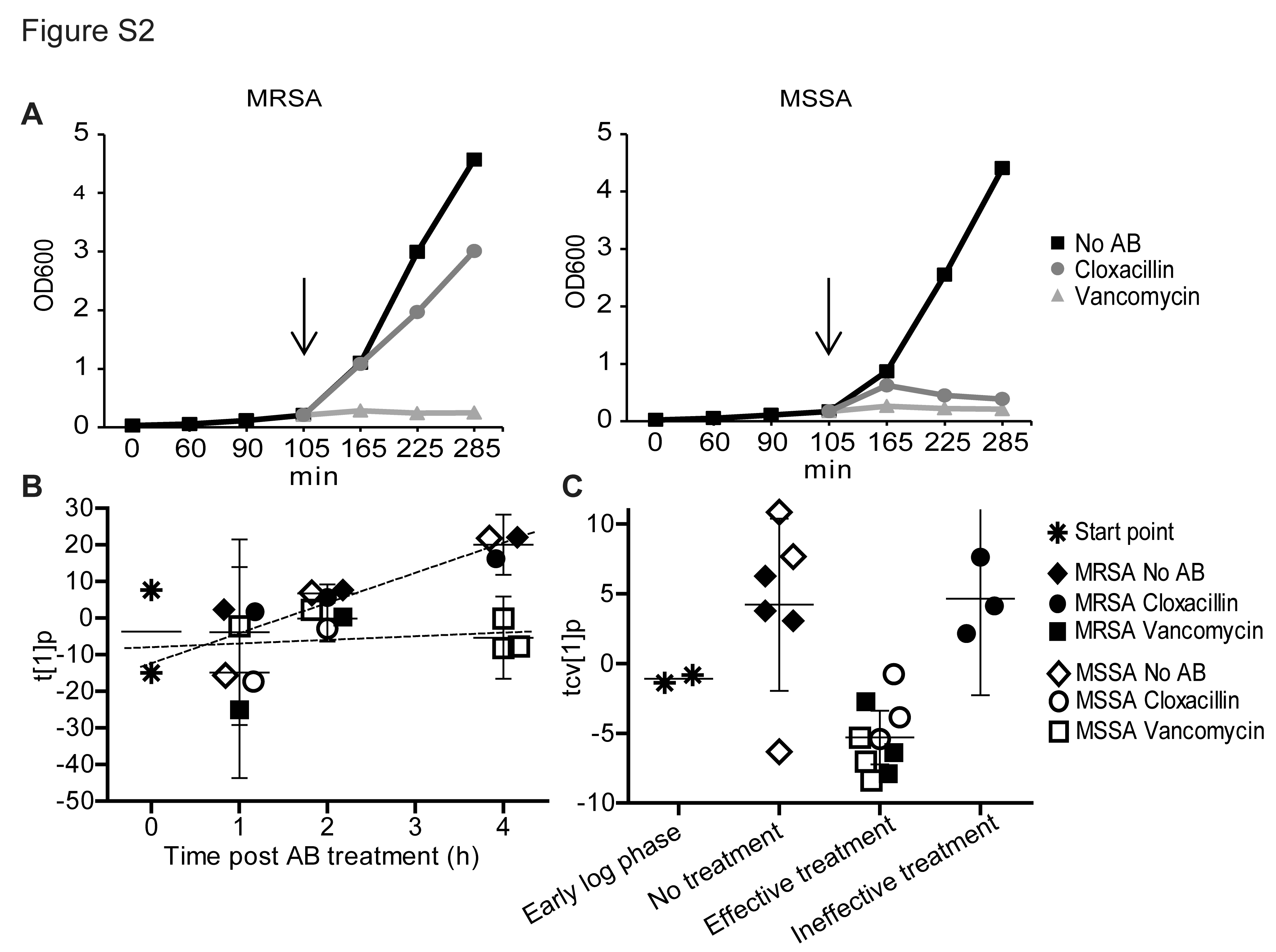

Supplement: Figure S2 — Metabolic profiles during in vitro growth of MRSA and MSSA. A) OD600 of MRSA and MSSA grown in absence of antibiotics, with vancomycin, or cloxacillin. Arrows indicate time point for addition of antibiotics. B) OPLS-DA predictive score vector, t [1] p, for a seven class model based on 367 metabolites showing bacterial growth and response to antibiotic treatment over time. Mean score values with 95% CI are shown. The two regression lines represent the direction over time for the two metabolic responses. C) Cross-validated OPLS-DA predictive score vector, tcv [1] p, for a two class model based on 367 metabolites revealing discrimination (p = 0.035) between the two responses. Mean score values with 95% CI are shown. (TIF) [file pone.0056971.s002.tif]
