# Supplementary material for: Metabolic Profiling for Detection of Staphylococcus aureus Infection and Antibiotic Resistance
Source: PLoS One. 2013 Feb 25;8(2):e56971. doi: 10.1371/journal.pone.0056971 (PMC3581498; doi:10.1371/journal.pone.0056971)
Supplement: Table S1 — Individual metabolite response to antibiotic treatment in vitro . (DOCX) [file pone.0056971.s003.docx]

**Supplementary Table 1. Individual metabolite response to antibiotic treatment *in vitro*.**

| Metabolite^a^ | Change in concentration with effective treatment^b^ | RI for three independent experiments^c^ | p-values for three independent experiments |
| --- | --- | --- | --- |
| 2-Aminobutyric acid | ↓ | 1182/1179/1181 | 2.2 x 10^-4^/ 3.0 x 10^-5^/ 8.9 x 10^-7^ |
| Alanine | ↑ | 1109/1105/1111 | 1.3 x 10^-3^/ 4.4 x 10^-3^/ 2.4 x 10^-5^ |
| Asparagine | ↑ | 1665/1664/1663 | 5.9 x 10^-14^/ 2.3 x 10^-5^/ 1.2 x 10^-11^ |
| Carbohydrate | ↑ | 2020/2019/2015 | 6.6 x 10^-5^/ 8.4 x 10^-3^/ 2.2 x 10^-4^ |
| Citric acid | ↑ | 1831/1831/1829 | 1.7 x 10^-3^/ 7.7 x 10^-4^/ 3.3 x 10^-4^ |
| Cysteine | ↓ | 1552/1551/1550 | 4.1 x 10^-2^/ 3.7 x 10^-2^/ 5.5 x 10^-4^ |
| Fructose | ↑ | 1866/1867/1863 | 5.2 x 10^-4^/ 3.7 x 10^-3^/ 9.2 x 10^-9^ |
| Glucose | ↑ | 1910/1910/1905 | 4.6 x 10^-2^/ 1.3 x 10^-2^/ 2.2 x 10^-9^ |
| Glutamic acid | ↑ | 1618/1616/1616 | 2.3 x 10^-3^/ 2.1 x 10^-2^/ 1.7 x 10^-2^ |
| Glutamine | ↑ | 1770/1769/1767 | 3.9 x 10^-9^/ 9.9 x 10^-8^/ 6.8 x 10^-17^ |
| Glycine | ↑ | 1308/1307/1306 | 2.9 x 10^-8^/ 2.2 x 10^-6^/ 5.7 x 10^-9^ |
| Homoserine | ↑ | 1454/1454/1453 | 1.4 x 10^-4^/ 2.1 x 10^-6^/ 1.9 x 10^-3^ |
| Inosine | ↑ | 2562/2562/2561 | 8.9 x 10^-4^/ 4.7 x 10^-5^/ 8.7 x 10^-6^ |
| Isoleucine | ↑ | 1298/1297/1295 | 3.0 x 10^-4^/ 3.4 x 10^-5^/ 7.9 x 10^-5^ |
| Malic acid | ↓ | 1486/1485/1484 | 5.8 x 10^-2^/ 7.6 x 10^-2^/ 1.2 x 10^-2^ |
| Mevalonic acid-1,5-lactone | ↓ | 1371/1366/1370 | 8.5 x 10^-2^/ 9.6 x 10^-2^/ 7.6 x 10^-3^ |
| Ornithine | ↑ | 1612/1611/1610 | 2.5 x 10^-6^/ 8.0 x 10^-3^/ 9.9 x 10^-3^ |
| Phenylalanine | ↑ | 1624/1623/1623 | 1.7 x 10^-4^/ 3.0 x 10^-4^/ 6.2 x 10^-3^ |
| Pyroglutamic acid | ↑ | 1523/1518/1522 | 8.7 x 10^-4^/ 2.7 x 10^-3^/ 2.7 x 10^-2^ |
| Ribitol | ↓ | 1719/1717/1714 | 7.2 x 10^-2^/ 6.5 x 10^-2^/ 7.6 x 10^-3^ |
| Ribose | ↓ | 1670/1670/1666 | 6.4 x 10^-2^/ 7.3 x 10^-2^/ 8.5 x 10^-3^ |
| Serine | ↑ | 1362/1360/1359 | 3.4 x 10^-15^/ 2.2 x 10^-11^/ 1.4 x 10^-14^ |
| Trehalose | ↑ | 2730/2730/2728 | 5.8 x 10^-3^/ 6.2 x 10^-3^/ 1.1 x 10^-4^ |
| Tryptophan | ↑ | 2207/2207/2207 | 1.9 x 10^-4^/ 9.2 x 10^-4^/ 6.2 x 10^-2^ |
| Tyrosine | ↑ | 1941/1940/1938 | 4.6 x 10^-3^/ 8.2 x 10^-3^/ 3.8 x 10^-3^ |
| Unid D | ↓ | 1380/1380/1379 | 1.1 x 10^-3^/ 4.6 x 10^-2^/ 1.1 x 10^-4^ |
| Unid I | ↑ | 1501/1500/1500 | 4.0 x 10^-13^/ 1.0 x 10^-9^/ 1.2 x 10^-12^ |
| Unid J | ↑ | 1631/1630/1629 | 7.0 x 10^-2^/ 2.1 x 10^-2^/ 2.0 x 10^-2^ |
| Unid K | ↑ | 1677/1677/1676 | 6.8 x 10^-5^/ 2.7 x 10^-6^/ 2.6 x 10^-8^ |
| Unid L | ↑ | 1761/1760/1758 | 1.1 x 10^-2^/ 5.1 x 10^-2^/ 1.6 x 10^-3^ |
| Unid P | ↑ | 2630/2630/2624 | 3.2 x 10^-2^/ 1.1 x 10^-2^/ 3.4 x 10^-6^ |
| Uric acid | ↑ | 2091/2091/2090 | 5.6 x 10^-3^/ 7.0 x 10^-2^/ 1.6 x 10^-2^ |

^a^Significant metabolites common between samples from the three independent experiments of *in vitro* grown MRSA and MSSA.

^b^Refers to response to antibiotic treatment, where ↑/↓ indicates a higher/lower metabolite concentration in samples with effective treatment compared to samples with ineffective treatment.

^c^Retention index for all metabolites.
