# Supplementary material for: Metabolic Profiling for Detection of Staphylococcus aureus Infection and Antibiotic Resistance
Source: PLoS One. 2013 Feb 25;8(2):e56971. doi: 10.1371/journal.pone.0056971 (PMC3581498; doi:10.1371/journal.pone.0056971)
Supplement: Table S2 — Individual metabolite response to antibiotic treatment in vivo . (DOCX) [file pone.0056971.s004.docx]

**Supplementary Table 2. Individual metabolite response to antibiotic treatment *in vivo*.**

| Metabolite^a^ | Change in concentration with effective treatment^b^ | RI^c^ | p-value^d^ | w*^e^ |
| --- | --- | --- | --- | --- |
| 1-Monohexadecanoylglycerol | ↓ | 2580 |  | * |
| 2,5-Diaminovalerolactam | ↓ | 1453 | * | * |
| 2-Aminomalonic acid | ↓ | 1465 | *** | * |
| 2-Monooleoylglycerol | ↑ | 2746 | * | * |
| 3-hydroxybutanoic acid | ↑ | 1172 | * | * |
| Aconitic acid | ↓ | 1743 |  | * |
| Alanine | ↓ | 1198 | ** | * |
| Alpha-Tocopherol | ↓ | 3138 | *** | * |
| Anthranilic acid | ↑ | 1609 | ** | * |
| Arginine | ↓ | 1819 | * | * |
| Carbohydrate | ↑ | 1950 | ** | * |
| Cholesterol | ↓ | 3155 | ** | * |
| Creatinine | ↓ | 1549 |  | * |
| Docosahexaenoic acid | ↑ | 2570 | ** | * |
| Erythritol | ↓ | 1501 |  | * |
| Erythrose | ↑ | 1420 | ** | * |
| Ethylamine | ↑ | 2153 | * | * |
| Fructose | ↓ | 1863 |  | * |
| Fucosterol | ↓ | 3243 |  | * |
| Fumaric acid | ↑ | 1351 |  | * |
| Gluconic acid | ↓ | 1956 | * | * |
| Glucose | ↑ | 1915 |  | * |
| Glutamine | ↓ | 1768 | * | * |
| Glutaric acid | ↑ | 1403 | ** | * |
| Glycerol | ↑ | 1277 |  | * |
| Glycerol-2-phosphate | ↓ | 1717 | ** | * |
| Glycerol-3-phosphate | ↓ | 1754 | *** | * |
| Glycine | ↑ | 1302 | * | * |
| Hexadecenoic acid | ↑ | 2022 | ** | * |
| Hydroxy-benzoic acid | ↓ | 1623 | *** | * |
| Inositol | ↓ | 2080 |  | * |
| Inositol-1-phosphate | ↓ | 2449 | * | * |
| Itaconic acid | ↓ | 1342 | * | * |
| Lauric acid | ↑ | 1650 | * | * |
| Linoleic acid | ↑ | 2208 |  | * |
| Lysine | ↓ | 1922 | ** | * |
| Malic acid | ↓ | 1483 | ** | * |
| Maltose | ↓ | 2725 | * | * |
| Mannose | ↑ | 1911 | * | * |
| Methionine | ↓ | 1515 |  | * |
| Methylglucopyranoside | ↑ | 2018 | * | * |
| Oleic acid | ↑ | 2214 | * | * |
| Ornithine | ↓ | 1610 |  | * |
| Pantothenic acid | ↓ | 1984 | * | * |
| Phenylalanine | ↓ | 1622 |  | * |
| Pseudouridine | ↑ | 2323 | ** | * |
| Ribitol | ↓ | 1696 |  | * |
| Serine | ↑ | 1358 | * | * |
| Succinic acid | ↑ | 1316 | * | * |
| Threitol | ↓ | 2487 |  | * |
| Threonic acid | ↓ | 1556 | * | * |
| Threonine | ↓ | 1383 | ** | * |
| Tyrosine | ↑ | 1937 | * | * |
| Unid 004 | ↓ | 1195 |  | * |
| Unid 007 | ↓ | 1214 | * | * |
| Unid 012 | ↓ | 1247 |  | * |
| Unid 018 | ↓ | 1338 | * | * |
| Unid 021 | ↑ | 1344 | * | * |
| Unid 028 | ↓ | 1367 | * | * |
| Unid 030 | ↑ | 1375 | ** | * |
| Unid 041 | ↓ | 1412 |  | * |
| Unid 044 | ↓ | 1436 |  | * |
| Unid 047 | ↓ | 1443 | * | * |
| Unid 048 | ↑ | 1445 | ** | * |
| Unid 049 | ↑ | 1451 | * | * |
| Unid 051 | ↓ | 1474 |  | * |
| Unid 058 | ↓ | 1528 |  | * |
| Unid 060 | ↓ | 1536 |  | * |
| Unid 062 | ↓ | 1557 |  | * |
| Unid 063 | ↓ | 1559 |  | * |
| Unid 065 | ↓ | 1568 | * | * |
| Unid 067 | ↑ | 1588 |  | * |
| Unid 068 | ↓ | 1592 | ** | * |
| Unid 069 | ↓ | 1594 | * | * |
| Unid 070 | ↑ | 1595 |  | * |
| Unid 071 | ↑ | 1596 | * | * |
| Unid 072 | ↓ | 1600 | * | * |
| Unid 076 | ↓ | 1626 | * | * |
| Unid 084 | ↑ | 1675 | *** | * |
| Unid 086 | ↓ | 1693 | * | * |
| Unid 088 | ↑ | 1702 | *** | * |
| Unid 089 | ↑ | 1708 |  | * |
| Unid 090 | ↑ | 1714 | ** | * |
| Unid 094 | ↓ | 1738 |  | * |
| Unid 096 | ↓ | 1757 | * | * |
| Unid 097 | ↑ | 1760 |  | * |
| Unid 098 | ↓ | 1763 |  | * |
| Unid 099 | ↑ | 1769 | ** | * |
| Unid 100 | ↓ | 1772 |  | * |
| Unid 102 | ↓ | 1779 |  | * |
| Unid 110 | ↓ | 1905 | *** | * |
| Unid 115 | ↓ | 1946 |  | * |
| Unid 117 | ↑ | 1952 | * | * |
| Unid 120 | ↑ | 1969 | ** | * |
| Unid 121 | ↑ | 1972 | * | * |
| Unid 124 | ↓ | 2017 |  | * |
| Unid 127 | ↑ | 2045 | * | * |
| Unid 130 | ↑ | 2059 | * | * |
| Unid 131 | ↑ | 2071 | ** | * |
| Unid 132 | ↑ | 2077 |  | * |
| Unid 135 | ↓ | 2105 | ** | * |
| Unid 136 | ↑ | 2107 | * | * |
| Unid 137 | ↑ | 2107 | *** | * |
| Unid 138 | ↓ | 2110 | *** | * |
| Unid 141 | ↑ | 2156 | ** | * |
| Unid 145 | ↓ | 2171 | * | * |
| Unid 146 | ↓ | 2174 |  | * |
| Unid 147 | ↓ | 2185 | ** | * |
| Unid 149 | ↓ | 2235 |  | * |
| Unid 151 | ↓ | 2248 | * | * |
| Unid 153 | ↓ | 2257 |  | * |
| Unid 154 | ↓ | 2275 |  | * |
| Unid 155 | ↓ | 2282 |  | * |
| Unid 157 | ↑ | 2336 |  | * |
| Unid 158 | ↓ | 2304 |  | * |
| Unid 159 | ↑ | 2327 |  | * |
| Unid 160 | ↑ | 2338 | * | * |
| Unid 161 | ↓ | 2346 | * | * |
| Unid 162 | ↓ | 2348 | * | * |
| Unid 163 | ↑ | 2396 | ** | * |
| Unid 164 | ↑ | 2403 |  | * |
| Unid 165 | ↑ | 2412 | * | * |
| Unid 166 | ↓ | 2415 | ** | * |
| Unid 167 | ↑ | 2423 |  | * |
| Unid 169 | ↓ | 2447 | * | * |
| Unid 171 | ↓ | 2486 | * | * |
| Unid 173 | ↓ | 2510 | * | * |
| Unid 175 | ↓ | 2540 |  | * |
| Unid 176 | ↓ | 2544 | ** | * |
| Unid 177 | ↓ | 2547 | *** | * |
| Unid 179 | ↓ | 2575 |  | * |
| Unid 181 | ↓ | 2594 | *** | * |
| Unid 182 | ↓ | 2598 |  | * |
| Unid 184 | ↑ | 2629 |  | * |
| Unid 186 | ↓ | 2665 |  | * |
| Unid 187 | ↓ | 2687 | *** | * |
| Unid 189 | ↑ | 2711 | ** | * |
| Unid 194 | ↑ | 2784 | ** | * |
| Unid 195 | ↓ | 2788 | ** | * |
| Unid 196 | ↓ | 2799 |  | * |
| Unid 198 | ↓ | 2826 |  | * |
| Unid 199 | ↓ | 2855 |  | * |
| Unid 200 | ↓ | 2866 |  | * |
| Unid 201 | ↓ | 2871 | ** | * |
| Unid 204 | ↓ | 2906 | *** | * |
| Unid 205 | ↓ | 2910 |  | * |
| Unid 206 | ↓ | 2938 |  | * |
| Unid 209 | ↑ | 2987 |  | * |
| Unid 210 | ↓ | 3002 |  | * |
| Unid 211 | ↓ | 3005 | * | * |
| Unid 214 | ↓ | 3033 | * | * |
| Unid 215 | ↓ | 3034 | * | * |
| Unid 224 | ↓ | 3217 |  | * |
| Unid 225 | ↓ | 3223 | *** | * |
| Unid 234 | ↓ | 3504 | ** | * |
| Unid 235 | ↓ | 3533 |  | * |
| Unid 237 | ↓ | 3570 |  | * |
| Unid 240 | ↓ | 3602 | ** | * |
| Unid 243 | ↓ | 3700 | *** | * |
| Unid 244 | ↓ | 3705 |  | * |
| Unid 245 | ↓ | 3724 | ** | * |
| Unid 247 | ↓ | 3748 | ** | * |
| Uracil | ↓ | 1336 | ** | * |
| Uric acid | ↓ | 2093 |  | * |
| Valine | ↓ | 1222 | ** | * |
| Xylose | ↑ | 1640 | * | * |
| Xylulose-5-phosphate | ↓ | 2109 |  | * |

^a^Significant metabolites in samples from mice infected with MRSA or MSSA.

^b^Refers to response to antibiotic treatment, where ↑/↓ indicates a higher/lower metabolite concentration in samples with effective treatment compared to samples with ineffective treatment.

^c^Retention index for all metabolites.

^d^Significance regarding p-values is stated with * for p < 0.05, ** for p < 0.01 and *** for p < 0.001.

^e^Significance regarding w* is stated with * for -0.04 > w* > 0.04.
