# Supplementary material for: Metabolic Profiling for Detection of Staphylococcus aureus Infection and Antibiotic Resistance
Source: PLoS One. 2013 Feb 25;8(2):e56971. doi: 10.1371/journal.pone.0056971 (PMC3581498; doi:10.1371/journal.pone.0056971)
Supplement: Table S3 — Individual metabolite response to antibiotic treatment common between in vitro grown MRSA and MSSA and mice infected with MRSA and MSSA. (DOCX) [file pone.0056971.s005.docx]

**Supplementary Table 3**. **Individual metabolite response to antibiotic treatment common between *in vitro* grown MRSA and MSSA and mice infected with MRSA and MSSA.**

| Metabolite^a^ | Change in concentration with effective treatment^b^ | Three independent *in vitro* experiments | | | | Mice infection | | |
| --- | --- | --- | --- | --- | --- | --- | --- | --- |
|  |  | **RI^c^** | **p-value^d^** | | **w*^e^** | **RI^c^** | **p-value^d^** | **w*^e^** |
| Carbohydrate | ↑ | 2020/2019/2015 | | ***/ ***/ *** | */*/* | 2018 | *** | * |
| Glucose | ↑ | 1910/1910/1905 | | */ */ *** | */*/* | 1915 | *** | * |
| Glycine | ↑ | 1308/1307/1306 | | ***/ ***/ *** | */*/* | 1302 | * | * |
| Malic acid | ↓ | 1486/1485/1484 | | / / * | */*/* | 1483 | *** | * |
| Ribitol | ↓ | 1719/1717/1714 | | / / ** | */*/* | 1711 |  | * |
| Serine | ↑ | 1362/1360/1359 | | ***/ ***/ *** | */*/* | 1358 | ** | * |
| Tyrosine | ↑ | 1941/1940/1938 | | **/ **/ ** | */*/* | 1937 | * | * |
| Unid L | ↑ | 1761/1760/1758 | | */ / ** | */*/* | 1760 |  | * |
| Unid P | ↑ | 2630/2630/2624 | | */ */ *** | */*/* | 2629 |  | * |

^a^Significant metabolites common between three in vitro experiments and one mouse experiment with MRSA and MSSA.

^b^Refers to response to antibiotic treatment, where ↑/↓ indicates a higher/lower metabolite concentration in samples with effective treatment compared to samples with ineffective treatment

^c^Retention index for all metabolites.

^d^Significance regarding p-values is stated with * for p < 0.05, ** for p < 0.01 and *** for p < 0.001.

^e^Significance regarding w* is stated with * for -0.04 > w* > 0.04.
