# Supplementary material for: Metabolic Profiling for Detection of Staphylococcus aureus Infection and Antibiotic Resistance
Source: PLoS One. 2013 Feb 25;8(2):e56971. doi: 10.1371/journal.pone.0056971 (PMC3581498; doi:10.1371/journal.pone.0056971)
Supplement: Table S4 — Metabolite changes of S. aureus infection that is common between samples from humans, mice, and in vitro . (DOCX) [file pone.0056971.s006.docx]

**Supplementary Table 4. Metabolite changes of *S. aureus* infection that is common between samples from humans, mice, and *in vitro*.**

| Metabolite^a^ | Change in concentration^b^ | RI^c^ | p-value^d^ | w*^e^ |
| --- | --- | --- | --- | --- |
| 3-hydroxybutanoic acid | ↓ | 1176 |  | * |
| Alpha-Tocopherol | ↑ | 3138 |  | * |
| Asparagine | ↑ | 1687 |  | * |
| Carbohydrate | ↑ | 2171 |  | * |
| Cholesterol | ↑ | 3152 |  | * |
| Cysteine | ↑ | 1552 |  | * |
| Erythritol | ↑ | 1503 |  | * |
| Glucose | ↑ | 1898 | * | * |
| Glutamine | ↓ | 1769 |  | * |
| Glycerol | ↓ | 1280 |  | * |
| Inositol | ↓ | 2081 |  | * |
| Inositol-1-phosphate | ↑ | 2401 | * | * |
| Linoleic acid | ↓ | 2208 |  | * |
| Lysine | ↓ | 1921 |  | * |
| Maltose | ↓ | 2724 | * | * |
| Methionine | ↑ | 1516 |  | * |
| Ornithine | ↓ | 1611 |  | * |
| Pseudouridine | ↑ | 2324 |  | * |
| Pyroglutamic acid | ↑ | 1520 |  | * |
| Ribitol | ↓ | 1697 |  | * |
| Ribose | ↑ | 1670 |  | * |
| Serine | ↑ | 1363 |  | * |
| Threonic acid | ↑ | 1538 |  | * |
| Threonine | ↑ | 1384 |  | * |
| Tryptophan | ↑ | 2206 |  | * |
| Unid Aa | ↓ | 2664 |  | * |
| Unid Ab | ↑ | 3004 |  | * |
| Unid C | ↓ | 1341 |  | * |
| Unid K | ↑ | 1677 |  | * |
| Unid O | ↓ | 2303 |  | * |
| Unid Y | ↓ | 1702 |  | * |
| Uric acid | ↓ | 2093 |  | * |
| Valine | ↑ | 1223 |  | * |

^a^Significant *S. aureus* metabolites common between *in vitro* experiments, mice infection, and human *S. aureus* or *E. coli* sepsis.

^b^Arrows indicate a change in metabolite concentration in human *S. aureus* sepsis samples compared to human *E. coli* sepsis samples.

^c^Retention index for all metabolites.

^d^Significance regarding p-values is stated with * for p < 0.05, ** for p < 0.01 and *** for p < 0.001.

^e^Significance regarding w* is stated with * for -0.04 > w* > 0.04.
