# Supplementary material for: Metabolic Profiling for Detection of Staphylococcus aureus Infection and Antibiotic Resistance
Source: PLoS One. 2013 Feb 25;8(2):e56971. doi: 10.1371/journal.pone.0056971 (PMC3581498; doi:10.1371/journal.pone.0056971)
Supplement: Table S5 — Individual metabolite response common between in vitro grown MRSA and MSSA and human S. aureus sepsis. (DOCX) [file pone.0056971.s007.docx]

**Supplementary Table 5**. **Individual metabolite response common between *in vitro* grown MRSA and MSSA and
human *S. aureus* sepsis.**

| Metabolite^a^ | Change in concentration with effective treatment^b^ | Three independent *in vitro* experiments | | | Human *S. aureus* sepsis | | |
| --- | --- | --- | --- | --- | --- | --- | --- |
|  |  | **RI^c^** | **p-values^d^** | **w*^e^** | **RI^c^** | **p-values^d^** | **w*^e^** |
| Alanine | ↑ | 1109/1105/1111 | **/ */ *** | */*/* | 1123 |  | * |
| Carbohydrate | ↑ | 2020/2019/2015 | ***/ ***/ *** | */*/* | 2020 |  | * |
| Cysteine | ↓ | 1552/1551/1550 | */ */ *** | */*/* | 1552 |  | * |
| Glutamine | ↑ | 1770/1769/1767 | ***/ ***/ *** | */*/* | 1769 | * | * |
| Homoserine | ↑ | 1454/1454/1453 | ***/ ***/ ** | */*/* | 1454 | * | * |
| Ornithine | ↑ | 1612/1611/1610 | ***/ **/ ** | */*/* | 1611 | * | * |
| Ribitol | ↓ | 1719/1717/1714 | //** | */*/* | 1712 |  | * |
| Ribose | ↓ | 1670/1670/1666 | //** | */*/* | 1670 |  | * |
| Serine | ↑ | 1362/1360/1359 | ***/ ***/ *** | */*/* | 1363 |  | * |
| Tryptophan | ↑ | 2207/2207/2207 | ***/ ***/ | */*/* | 2206 |  | * |
| Unid D | ↓ | 1380/1380/1379 | **/ **/ ** | */*/* | 1380 |  | * |
| Unid J | ↑ | 1631/1630/1629 | /*/ * | */*/* | 1630 |  | * |
| Uric acid | ↑ | 2091/2091/2090 | **/ / * | */*/* | 2093 |  | * |

^a^Significant metabolites common between samples from human patients with severe sepsis caused by *S. aureus* and samples from the three independent analyses of *in vitro* growth of MRSA and MSSA.

^b^Refers to response to antibiotic treatment, where ↑/↓ indicates a higher/lower metabolite concentration in samples with effective treatment compared to samples with ineffective treatment (for *in vitro* experiments) and in late time point, 144h-2weeks after admittance, compared to acute phase infection samples, 0-24h after admittance (for human sepsis).

^c^Retention index for all metabolites.

^d^Significance regarding p-values is stated with * for p < 0.05, ** for p < 0.01 and *** for p < 0.001.

^e^Significance regarding w* is stated with * for -0.04 > w* > 0.04.
