# Supplementary material for: Metabolic Profiling for Detection of Staphylococcus aureus Infection and Antibiotic Resistance
Source: PLoS One. 2013 Feb 25;8(2):e56971. doi: 10.1371/journal.pone.0056971 (PMC3581498; doi:10.1371/journal.pone.0056971)
Supplement: Table S6 — Individual metabolite response common between mice infected with MRSA and MSSA and human S. aureus sepsis. (DOCX) [file pone.0056971.s008.docx]

**Supplementary Table 6**. **Individual metabolite response common between mice infected with MRSA and MSSA and human *S. aureus* sepsis.**

| Metabolite^a^ | Change in concentration with effective treatment^b^ | Human *S. aureus* sepsis | | | Mice infection | | |
| --- | --- | --- | --- | --- | --- | --- | --- |
|  |  | **RI^c^** | **p-values^d^** | **w*^e^** | **RI^c^** | **p-values^d^** | **w*^e^** |
| 3-hydroxybutanoic acid | ↑ | 1176 |  | * | 1172 | * | * |
| Carbohydrate | ↑ | 1951 |  | * | 1950 | *** | * |
| Cholesterol | ↓ | 3152 |  | * | 3155 | ** | * |
| Creatinine | ↓ | 1549 |  | * | 1549 |  | * |
| Erythritol | ↓ | 1503 |  | * | 1501 |  | * |
| Erythrose | ↑ | 1450 |  | * | 1449 | ** | * |
| Glycerol | ↑ | 1280 |  | * | 1277 | ** | * |
| Glycerol-3-phosphate | ↓ | 1753 |  | * | 1754 | *** | * |
| Inositol | ↓ | 2081 |  | * | 2080 |  | * |
| Linoleic acid | ↑ | 2208 |  | * | 2208 |  | * |
| Ribitol | ↓ | 1712 |  | * | 1696 |  | * |
| Serine | ↑ | 1363 |  | * | 1358 | ** | * |
| Threonic acid | ↓ | 1538 |  | * | 1556 |  | * |
| Threonine | ↓ | 1384 |  | * | 1383 | ** | * |
| Unid Y | ↑ | 1702 |  | * | 1702 | *** | * |

^a^Significant metabolites common between samples from human patients with severe sepsis caused by *S. aureus* and samples from the analysis of mice infected with *S. aureus*. (A metabolite is considered significant with either a 0.04 > w* > 0.04 or a p-value < 0.05.)

^b^Refers to response to antibiotic treatment, where ↑/↓ indicates a higher/lower metabolite concentration in samples with effective treatment compared to samples with ineffective treatment (for mice infection) and in late time point, 144h-2weeks after admittance, compared to acute phase infection samples, 0-24h after admittance (for human sepsis).

^c^Retention index for all metabolites.

^d^Significance regarding p-values is stated with * for p < 0.05, ** for p < 0.01 and *** for p < 0.001.

^e^Significance regarding w* is stated with * for -0.04 > w* > 0.04.
