# Supplementary material for: Metabolic Profiling for Detection of Staphylococcus aureus Infection and Antibiotic Resistance
Source: PLoS One. 2013 Feb 25;8(2):e56971. doi: 10.1371/journal.pone.0056971 (PMC3581498; doi:10.1371/journal.pone.0056971)
Supplement: Table S7 — Individual metabolite response common between human S. aureus sepsis and mice infected with MRSA and MSSA, and human S. aureus sepsis and in vitro grown MRSA and MSSA. (DOCX) [file pone.0056971.s009.docx]

**Supplementary Table 7. Individual metabolite response common between human *S. aureus* sepsis and mice infected with MRSA and
MSSA, and human *S. aureus* sepsis and in vitro grown MRSA and MSSA.**

| Metabolite^a^ | Change in concentration with effective treatment^b^ | Three independent *in vitro* experiments | | | Mice infection | | | Human *S. aureus* sepsis | | |
| --- | --- | --- | --- | --- | --- | --- | --- | --- | --- | --- |
|  |  | **RI^c^** | **p-values^d^** | **w*^e^** | **RI^c^** | **p-values^d^** | **w*^e^** | **RI^c^** | **p-values^d^** | **w*^e^** |
| 3-hydroxy-butanoic acid | ↑ |  |  |  | 1172 | * | * | 1176 |  | * |
| Alanine | ↑ | 1109/1105/1111 | **/ **/ *** | */*/* |  |  |  | 1123 |  | * |
| Carbohydrate | ↑ | 2020/2019/2015 | ***/ ***/ *** | */*/* | 1950 | *** | * | 2020 |  | * |
| Cholesterol | ↓ |  |  |  | 3155 | ** | * | 3152 |  | * |
| Creatinine | ↓ |  |  |  | 1549 |  | * | 1549 |  | * |
| Cysteine | ↓ | 1552/1551/1550 | */ */ *** | */*/* |  |  |  | 1552 |  | * |
| Erythritol | ↓ |  |  |  | 1501 |  | * | 1503 |  | * |
| Erythrose | ↑ |  |  |  | 1449 | ** | * | 1450 |  | * |
| Glutamine | ↑ | 1770/1769/1767 | ***/ ***/ *** | */*/* |  |  |  | 1769 | * | * |
| Glycerol | ↑ |  |  |  | 1277 | ** | * | 1280 |  | * |
| Glycerol-3-phosphate | ↓ |  |  |  | 1754 | *** | * | 1753 |  | * |
| Homoserine | ↑ | 1454/1454/1453 | ***/***/ ** | */*/* |  |  |  | 1454 | * | * |
| Inositol | ↓ |  |  |  | 2080 |  | * | 2081 |  | * |
| Linoleic acid | ↑ |  |  |  | 2208 |  | * | 2208 |  | * |
| Ornithine | ↑ | 1612/1611/1610 | ***/ **/ ** | */*/* |  |  |  | 1611 | * | * |
| Ribitol | ↓ | 1719/1717/1714 | / / ** | */*/* | 1696 |  | * | 1712 |  | * |
| Ribose | ↓ | 1670/1670/1666 | / / ** | */*/* |  |  |  | 1670 |  | * |
| Serine | ↑ | 1362/1360/1359 | ***/ ***/ *** | */*/* | 1358 | ** | * | 1363 |  | * |
| Threonic acid | ↓ |  |  |  | 1556 |  | * | 1538 |  | * |
| Threonine | ↓ |  |  |  | 1383 | ** | * | 1384 |  | * |
| Tryptophan | ↑ | 2207/2207/2207 | ***/ ***/ | */*/* |  |  |  | 2206 |  | * |
| Unid D | ↓ | 1380/1380/1379 | **/ **/** | */*/* |  |  |  | 1380 |  | * |
| Unid J | ↑ | 1631/1630/1629 | / */ * | */*/* |  |  |  | 1630 |  | * |
| Unid Y | ↑ |  |  |  | 1702 | *** | * | 1702 |  | * |
| Uric acid | ↑ | 2091/2091/2090 | **/ / * | */*/* |  |  |  | 2093 |  | * |

^a^Significant metabolites common between human and *in vitro* samples and human and mice samples. (A metabolite is considered significant with either a 0.04 >w* > 0.04 or a p-value <0.05.)

^b^Refers to response to antibiotic treatment, where ↑/↓ indicates a higher/lower metabolite concentration in samples with effective treatment compared to samples with ineffective treatment (for *in vitro* experiments and mice infection) and in late time point, 144h-2weeks after admittance, compared to acute phase infection samples, 0-24h after admittance (for human sepsis).

^c^Retention index for all metabolites.

^d^Significance regarding p-values is stated with * for p <0.05, ** for p <0.01 and *** for p <0.001.

^e^Significance regarding w* is stated with * for -0.04 >w* >0.04.
