# Supplementary material for: Metabolic Profiling for Detection of Staphylococcus aureus Infection and Antibiotic Resistance
Source: PLoS One. 2013 Feb 25;8(2):e56971. doi: 10.1371/journal.pone.0056971 (PMC3581498; doi:10.1371/journal.pone.0056971)
Supplement: Table S8 — Summary of characteristic and high intensity m/z values for the different sets of common metabolites. (DOCX) [file pone.0056971.s010.docx]

**Supplementary Table 8. Summary of characteristic and high intensity m/z values for the different sets of common metabolites.**

| Name^a^ | TMS^b^ | M^●+c^ | m/z 1^d^ | m/z 2 | m/z 3 | m/z 4 | m/z 5 | m/z 6 | m/z 7 | m/z 8 | m/z 9 | m/z 10 |
| --- | --- | --- | --- | --- | --- | --- | --- | --- | --- | --- | --- | --- |
| 2-Amino-butyric acid | 2 | 247 (0) | 232 (<1) | 218 (1) | 204 (2) | 188 (3) | 147 (13) | 131 (11) | 130 (74) | 114 (2) | 100 (6) | 73 (100) |
| 3-hydroxy-butanoic acid | 2 | 248 (0) | 233  (9) | 191 (20) | 148 (15) | 147 (97) | 133 (11) | 117 (45) | 115 (7) | 88 (15) | 75 (52) | 73 (100) |
| Alanine | 2 | 233 (0) | 218  (1) | 202 (1) | 190 (4) | 174 (8) | 158 (<1) | 147 (20) | 117 (13) | 116 (99) | 100 (8) | 73 (100) |
| Alpha-Tocopherol | 1 | 502 (17) | 502 (17) | 277 (4) | 238 (13) | 237 (54) | 236 (32) | 221 (5) | 209 (2) | 208 (5) | 193 (3) | 73 (100) |
| Asparagine | 3 | 348 (<1) | 348 (<1) | 258 (2) | 231 (12) | 202 (3) | 188 (10) | 147 (11) | 141 (10) | 132 (20) | 116 41) | 73 (100) |
| Carbohydrate |  |  | 319  (4) | 305 (<1) | 220 (4) | 217 (5) | 204 (21) | 147 (13) | 129 (4) | 103 (5) | 89  (5) | 73 (100) |
| Cholesterol | 1 | 458 (5) | 458  (5) | 368 (12) | 329 (10) | 159 (17) | 145 (26) | 129 (94) | 119 (31) | 105 (40) | 91 (44) | 73 (100) |
| Citric acid | 4 | 480 (0) | 465  (1) | 375 (3) | 347 (3) | 305 (1) | 273 (18) | 211 (2) | 183 (4) | 147 (32) | 133 (6) | 73 (100) |
| Creatinine | 3 | 329 (10) | 329 (10) | 314 (4) | 241 (2) | 187 (3) | 171 (15) | 147 (8) | 143 (48) | 115 (97) | 100 31) | 73 (100) |
| Cysteine | 3 | 337 (0) | 322 (<1) | 294 (<1) | 220 (29) | 218 (26) | 204 (2) | 147 (24) | 132 (8) | 116 (6) | 100 24) | 73 (100) |
| Erythritol | 4 | 410 (0) | 320 (<1) | 287 (3) | 217 (14) | 189 (4) | 147 (22) | 133 (7) | 117 (11) | 103 (13) | 89  (8) | 73 (100) |
| Erythrose | 3 | 365 (<1) | 365 (<1) | 350 (9) | 262 (3) | 221 (3) | 148 (6) | 147 (35) | 133 (9) | 117 (5) | 100 (3) | 73 (100) |
| Fructose | 5 | 569 (<1) | 569 (<1) | 364 (2) | 335 (1) | 307 (14) | 217 (41) | 189 (5) | 147 (24) | 117 (8) | 103 (74) | 73 (100) |
| Glucose | 5 | 569 (0) | 464 (<1) | 319 (6) | 217 (9) | 205 (18) | 160 (21) | 147 (26) | 129 (8) | 117 (10) | 103 18) | 73 (100) |
| Glutamic acid | 3 | 363 (1) | 363  (1) | 246 (43) | 230 (7) | 218 (4) | 156 (17) | 147 (26) | 140 (3) | 133 (9) | 128 28) | 73 (100) |
| Glutamine | 3 | 362 (<1) | 362 (<1) | 347 (1) | 245 (7) | 229 (2) | 203 (4) | 156 (62) | 155 (18) | 147 (13) | 75 (31) | 73 (100) |
| Glycerol | 3 | 308 (0) | 293 (<1) | 218 (14) | 206 (5) | 205 (27) | 148 (10) | 147 (61) | 133 (19) | 117 (36) | 103 (29) | 73 (100) |
| Glycerol-3-phosphate | 4 | 460 (0) | 445  (2) | 357 (9) | 315 (4) | 300 (4) | 299 (15) | 147 (14) | 133 (11) | 103 (10) | 101 12) | 73 (100) |
| Glycine | 3 | 291 (0) | 276  (4) | 248 (13) | 202 (<1) | 175 (17) | 174 (89) | 147 (33) | 133 (15) | 100 (22) | 86 (39) | 73 (100) |
| Homoserine | 3 | 335 (0) | 320 (<1) | 292 (<1) | 219 (7) | 218 (30) | 202 (2) | 147 (11) | 128 (26) | 103 (22) | 100 (7) | 73 (100) |
| Inosine | 4 | 556 (0) | 541 (<1) | 281 (4) | 259 (3) | 245 (6) | 230 (9) | 217 (10) | 193 (6) | 147 (9) | 103 10) | 73 (100) |
| Inositol | 6 | 612 (<1) | 612 (<1) | 507 (<1) | 318 (12) | 305 (14) | 265 (6) | 217 (33) | 204 (6) | 191 (19) | 147 42) | 73 (100) |
| Inositol-1-phosphate | 7 | 764 (0) | 387  (4) | 373 (<1) | 318 (9) | 315 (8) | 299 (8) | 217 (3) | 191 (4) | 147 (21) | 75  (9) | 73 (100) |
| Isoleucine | 2 | 275 (0) | 260  (1) | 232 (4) | 218 (16) | 159 (13) | 158 (85) | 147 (14) | 142 (11) | 100 (13) | 75 (11) | 73 (100) |
| Linoleic acid | 1 | 352 (<1) | 352 (<1) | 337 (5) | 135 (8) | 129 (25) | 117 (20) | 109 (10) | 95 (23) | 81 (41) | 75 (100) | 73  (75) |
| Lysine | 4 | 434 (1) | 434  (1) | 329 (1) | 317 (8) | 230 (10) | 174 (41) | 156 (50) | 128 (16) | 100 (13) | 86 (15) | 73 (100) |
| Malic acid | 3 | 350 (0) | 335  (1) | 307 (<1) | 245 (3) | 233 (5) | 217 (1) | 189 (3) | 175 (4) | 147 (30) | 133 (9) | 73 (100) |
| Maltose | 8 | 947 (0) | 464 (<1) | 338 (5) | 217 (3) | 205 (2) | 169 (3) | 147 (14) | 129 (6) | 117 (6) | 103 (8) | 73 (100) |
| Methionine | 2 | 293 (2) | 293  (2) | 250 (2) | 232 (6) | 219 (3) | 202 (2) | 188 (2) | 176 (33) | 147 (16) | 128 (31) | 73 (100) |
| Mevalonic acid-1,5-lactone | 1 | 202 (0) | 187 (11) | 157 (5) | 145 (70) | 143 (25) | 115 (72) | 103 (6) | 101 (19) | 99  (7) | 75 (100) | 73  (56) |
| Ornithine | 3 | 348 (2) | 348  (2) | 204 (3) | 162 (4) | 147 (10) | 142 (45) | 128 (6) | 115 (8) | 102 (10) | 73 100) | 70  (55) |
| Phenylalanine | 2 | 309 (0) | 294 (<1) | 219 (7) | 218 (35) | 192 (19) | 160 (1) | 147 (12) | 130 (3) | 100 (20) | 91  (9) | 73 (100) |
| Pseudouridine | 5 | 604 (0) | 589 (<1) | 496 (<1) | 424 (2) | 357 (4) | 269 (2) | 217 (32) | 147 (12) | 133 (4) | 103 (6) | 73 (100) |
| Pyroglutamic acid | 2 | 273 (0) | 264  (2) | 258 (4) | 230 (5) | 214 (<1) | 161 (42) | 156 (58) | 147 (16) | 133 (4) | 75 (13) | 73 (100) |
| Ribitol | 5 | 512 (0) | 352 (<1) | 319 (3) | 307 (2) | 217 (17) | 205 (6) | 147 (21) | 129 (8) | 117 (7) | 103 (19) | 73 (100) |
| Ribose | 4 | 467 (0) | 307  (4) | 277 (1) | 217 (14) | 189 (5) | 160 (3) | 147 (15) | 133 (6) | 117 (5) | 103 (36) | 73 (100) |
| Serine | 3 | 321 (0) | 306  (2) | 278 (3) | 218 (31) | 204 (38) | 188 (7) | 147 (18) | 133 (8) | 116 (7) | 100 (25) | 73 (100) |
| Threonic acid | 4 | 424 (0) | 409 (<1) | 245 (8) | 220 (6) | 217 (6) | 205 (4) | 147 (35) | 133 (6) | 117 (10) | 103 (8) | 73 (100) |
| Threonine | 3 | 335 (0) | 320 (<1) | 291 (2) | 219 (9) | 218 (8) | 147 (8) | 129 (5) | 117 (16) | 101 (14) | 75 (14) | 73 (100) |
| Trehalose | 8 | 918 (0) | 326  (5) | 361 (12) | 243 (3) | 217 (9) | 191 (15) | 169 (7) | 147 (20) | 129 (12) | 103 (16) | 73 (100) |
| Tryptophan | 3 | 420 (0) | 405 (<1) | 377 (<1) | 291 (4) | 218 (2) | 203 (11) | 202 (54) | 147 (4) | 100 (4) | 75 (10) | 73 (100) |
| Tyrosine | 3 | 397 (0) | 382  (1) | 354 (2) | 280 (8) | 220 (9) | 219 (19) | 218 (100) | 179 (9) | 100 (26) | 75  (4) | 73  (19) |
| Unid Aa |  |  | 321  (3) | 255 (2) | 217 (8) | 201 (1) | 174 (7) | 159 (29) | 147 (14) | 117 (10) | 103 (17) | 73 (100) |
| Unid Ab |  |  | 370  (5) | 315 (9) | 299 (23) | 243 (17) | 227 (18) | 211 (21) | 155 (11) | 145 (9) | 129 (75) | 73 (100) |
| Unid D |  |  | 240  (2) | 224 (3) | 191 (7) | 169 (14) | 155 (36) | 140 (5) | 126 (4) | 99 (94) | 69 (88) | 56 (100) |
| Unid I |  |  | 287 (<1) | 243 (30) | 201 (4) | 158 (5) | 142 (4) | 128 (49) | 115 (78) | 100 (100) | 73 (61) | 59  (23) |
| Unid J |  |  | 292 (<1) | 258 (<1) | 218 (2) | 204 (17) | 160 (10) | 147 (18) | 117 (4) | 100 (7) | 75 (21) | 73 (100) |
| Unid K |  |  | 345  (2) | 317 (2) | 234 (<1) | 174 (2) | 147 (21) | 142 (3) | 133 (9) | 100 (7) | 75 (26) | 73 (100) |
| Unid L |  |  | 450 (<1) | 285 (<1) | 230 (2) | 218 (15) | 217 (42) | 189 (2) | 147 (9) | 100 (7) | 75 (16) | 73 (100) |
| Unid O |  |  | 417 (<1) | 269 (4) | 246 (4) | 232 (2) | 217 (5) | 147 (16) | 133 (4) | 103 (16) | 73 (100) | 56  (76) |
| Unid P |  |  | 458 (<1) | 367 (<1) | 262 (<1) | 220 (4) | 206 (2) | 174 (3) | 147 (10) | 132 (6) | 104 (10) | 73 (100) |
| Unid Y |  |  | 406 (<1) | 304 (4) | 233 (2) | 163 (3) | 156 (5) | 147 (14) | 133 (3) | 105 (4) | 84 (20) | 73 (100) |
| Uric acid | 4 | 456 (8) | 456  (8) | 442 (4) | 441 (7) | 382 (5) | 367 (3) | 353 (2) | 147 (17) | 131 (4) | 100 (7) | 73 (100) |
| Valine | 2 | 261 (0) | 246 (<1) | 218 (10) | 160 (<1) | 156 (2) | 147 (14) | 144 (64) | 114 (2) | 100 (11) | 75 (12) | 73 (100) |

^a^The table includes metabolites from the different set of common metabolites represented in tables S1-S7 with the exception of table S2.

^b^States the number of trimethylsilyl (TMS) groups of the derivatized form of the identified metabolites from which the m/z values are obtained.

^c^Mass to charge ratio (m/z) for the molecular radical cation M^●+^ followed by the relative intensity in percent within parenthesis. A relative intensity value of 0 indicates that the molecular radical cation is not present in the mass spectrum. No molecular radical cation can be given for the unidentified metabolites.

^d^m/z 1-10 represents the mass to charge ratio of ten characteristic and high intensity ions in the metabolite mass spectrum followed by the relative intensity of the peak in percent within parenthesis. The relative intensities varies between <1 % for low abundant peaks and 100 % for the highest peak. The ions are listed in order of decreasing m/z.
